# Thymic Function as a Predictor of Immune Recovery in Chronically HIV-Infected Patients Initiating Antiretroviral Therapy

**DOI:** 10.3389/fimmu.2019.00025

**Published:** 2019-02-05

**Authors:** Rita Rb-Silva, Claudia Nobrega, Cecilia Azevedo, Emilia Athayde, João Canto-Gomes, Ivo Ferreira, Rémi Cheynier, Andrew J. Yates, Ana Horta, Margarida Correia-Neves

**Affiliations:** ^1^Population Health Research Domain, Life and Health Sciences Research Institute, School of Medicine, University of Minho, Braga, Portugal; ^2^ICVS/3B's, PT Government Associate Laboratory, Braga/Guimarães, Portugal; ^3^Department of Onco-Hematology, Portuguese Institute of Oncology of Porto, Porto, Portugal; ^4^Department of Mathematics and Applications, School of Sciences, University of Minho, Braga, Portugal; ^5^Center of Mathematics, University of Minho, Braga, Portugal; ^6^INSERM, U1016, Institut Cochin, Paris, France; ^7^CNRS, UMR8104, Paris, France; ^8^Department of Infection, Immunity and Inflammation, Université Paris Decartes, Paris, France; ^9^Department of Pathology & Cell Biology, Columbia University, New York, NY, United States; ^10^Department of Infectious Diseases, Centro Hospitalar do Porto, Porto, Portugal; ^11^Division of Infectious Diseases, Department of Medicine Solna, Karolinska Institutet, Stockholm, Sweden

**Keywords:** poor immunological responders, predictive modeling, immune recovery, CD4^+^ T cells, thymic function, immune activation, antiretroviral therapy, HIV infection

## Abstract

Poor immunological responders (PIR) are HIV-infected patients with virologic suppression upon antiretroviral therapy (ART) but persistently low CD4^+^ T cell counts. Early identification of PIR is important given their higher morbimortality compared to adequate immune responders (AIR). In this study, 33 patients severely lymphopenic at ART onset, were followed for at least 36 months, and classified as PIR or AIR using cluster analysis grounded on their CD4^+^ T cell count trajectories. Based on a variety of immunological parameters, we built predictive models of PIR/AIR outcome using logistic regression. All PIR had CD4^+^ T cell counts consistently below 500 cells/μL, while all AIR reached this threshold. AIR showed a higher percentage of recent thymic emigrants among CD4^+^ T cells; higher numbers of sj-TRECs and greater sj/β TREC ratios; and significant increases in thymic volume from baseline to 12 months of ART. We identified mathematical models that correctly predicted PIR/AIR outcome after 36 months of therapy in 77–87% of the cases, based on observations made until 2–6 months after ART onset. This study highlights the importance of thymic activity in the immune recovery of severely lymphopenic patients, and may help to select the patients that will benefit from closer follow-up or novel therapeutic approaches.

## Introduction

The majority of patients infected by the human immunodeficiency virus (HIV) show immune recovery upon antiretroviral therapy (ART). This recovery usually occurs with a rapid increase in CD4^+^ T cell numbers during the first few months, followed by a slower increase toward a plateau (1–3). An adequate immune recovery is defined as the attainment of CD4^+^ T cell counts within the range observed in healthy adult individuals (i.e., 500–1,500 cells/μL). However, CD4^+^ T cell counts remain persistently low in 9–45% of patients, depending on the criteria used, despite complete viral suppression (i.e., undetectable plasma HIV RNA) (4–6). There is no consensual case-definition for these patients, and dozens of different terms are used in the literature to refer to them, such as “poor immunological responders”, “immunological non-responders”, and “discordant immune responders” (5). In this study, we use the terms poor and adequate immunological responders (PIR and AIR), because these seem to be the terms that best reflect patients' immunologic response to therapy.

Distinct immune recovery trajectories throughout ART result from differences in CD4^+^ T cell production by the thymus, peripheral proliferation, virus- or immune-mediated cell death and/or ability to obtain survival stimuli. Differences in recovery may also derive from variations in the extent of CD4^+^ T cell migration from lymphoid tissues to peripheral blood or to the gastrointestinal tract soon after ART initiation (1, 7, 8).

Production of new T cells by the thymus appears to be a major driver of immune recovery during ART, being particularly relevant for patients who start therapy with <200 CD4^+^ T cells/μL, in whom poor immune responses are more frequent (9–16). Many other factors have been associated with poor immune recovery, such as older age, lower nadir CD4^+^ T cell counts, residual viral replication, increased T cell death, immune hyperactivation, altered ratio of regulatory T cells (Treg) to Th17 cells, tissue fibrosis [reviewed in (17, 18)] and specific metabolic profiles (19).

PIR have higher morbidity and mortality rates than AIR (20–22). Therefore, there is an urgent need for tools to identify PIR early and improve their prognoses. This study discriminated PIR and AIR among patients with acquired immunodeficiency syndrome (AIDS) and <200 CD4^+^ T cells/μL at ART onset, not using any of the many clinical criteria reported in the literature, but instead using clustering analysis of longitudinal data. For this analysis, we simply specified that patients should be divided into 2 clusters on the basis of their CD4^+^ T cell count trajectories. *A posteriori* analysis of the trajectories revealed that one cluster included patients whose trajectories reached higher CD4^+^ T cell counts, with all patients in that cluster presenting > 500 CD4^+^ T cells/μL at least at one time point over the first 36 months of therapy (AIR), contrary to patients of the other cluster (PIR). In addition, immunological parameters were compared between PIR and AIR and early alterations were identified as predictors of PIR status after 36 months of therapy.

## Materials and Methods

### Study Participants

Patients infected by HIV, with <200 CD4^+^ T cells/μL at ART initiation and with ≥36 months of follow-up were selected (*n* = 33) from a prospective cohort of patients (*n* = 100 individuals; Figure S1) on medical care at the Centro Hospitalar do Porto, Portugal. The enrolment period ran between April 2010 and October 2012. All patients were provided an explanation of the study and signed an informed consent (local Ethical Committee approval—reference 168/CES); were older than 18 years, chronically infected with HIV-1, ART-naïve at enrolment and with clinical criteria to initiate ART. ART schemes chosen for each individual took into consideration national and international guidelines. All patients were therapy compliant throughout the follow-up; after a median time of 6 months of ART, all patients presented sustained plasma viral loads below 50 copies/mL, except for 4 individuals who had viral blips (Figure S2). Clinical information and peripheral blood samples were retrieved at baseline (just before ART initiation) and at 2, 6, 12, 16, 20, 24, 28, 32, 36, 42, 48, 54, and 60 months of ART (median time deviations to each time point was ≤ 8 days). Individuals were followed for at least 36 months, with median follow-up time of 60 months. CD4^+^ T cell counts and plasma viral load quantification were assessed at all available time points by a certified laboratory.

### Imaging

Sixteen of the 33 patients underwent chest computed tomography (CT) scans at baseline and at 12 months of ART (Figure S1). CT scans were performed without contrast in a Siemens Somatom emotion apparatus (16 sections). Thymic volume was considered as the mean of measurements, blindly performed by two independent operators, in cm^3^. Thymic index, assessed by one of the operators, was determined by scoring the presence of thymic tissue as opposed to adipose tissue: (0) thymus entirely replaced by fat; (1) minimal, barely recognizable, soft tissue; (2) minimal, but more obvious, soft tissue; (3) moderate soft tissue; (4) moderate soft tissue of greater extent, almost mass like; (5) mass-like appearance that raises concern for a thymoma (23). Both operators were blinded to any demographic or clinical data besides the HIV serostatus.

### Blood Processing and Flow Cytometry (FACS) Analysis

For each participant and at each time point, venous blood was collected to K_2_EDTA collecting tubes and processed on the same day. A blood aliquot for FACS analysis was taken and, from the remaining blood, PBMCs were isolated by gradient centrifugation using Histopaque 1077 (Sigma-Aldrich, United Kingdom). After PBMCs' enumeration, 2 × 10^6^ cells were used for FACS staining and 1 × 10^6^ cells aliquots were stored at −80 °C for TRECs quantification.

For FACS, three antibody panels were design for evaluation of T cell activation (Panel 1, performed in 100 μL of whole blood), recent thymic emigrants (RTE; Panel 2, performed in 200 μL of whole blood) and Treg (Panel 3, performed in 2 × 10^6^ PBMCs), as previously described (24). A mixture of anti-CD45RA-FITC (HI100), anti-CD69-PE (FN50), anti-CD45RO-PerCP/Cy5.5 (UCHL1), anti-HLA-DR-PeCy7 (L243), anti-CD8-APC (RPA-T8), anti-CD4-APC/Cy7 (RPA-T4), and anti-CD3-Pacific Blue (OKT3) was used for Panel 1; a mixture of anti-CD45RA-FITC (HI100), anti-CD3-PE (OKT3), anti-CD45RO-PerCP/Cy5.5 (UCHL1); anti-CD31-PeCy7 (WM59), anti-CCR7-Alexa Fluor 647 (G043H7), anti-CD4-APC/Cy7 (RPA-T4), and anti-CD8-Brilliant Violet 421 (RPA-T8) was used for Panel 2; and a mixture of anti-Ki67-FITC (MOPC-21), anti-FoxP3-PE (PCH101), anti-CD127-PerCP/Cy5.5 (AO19D5), anti-CD31-PeCy7 (WM59), anti-CD25-APC (BC96), anti-CD4-APC/Cy7 (RPA-T4), anti-CD45RA-Pacific Blue (HI100), and anti-CD3-V500 (UCHT1) was used for Panel 3. All antibodies were from BioLegend, except for anti-CD3-V500 (BD Horizon), anti-FoxP3-PE (eBiosciences) and anti-Ki67 (BD Pharmigen). Samples were acquired using a BD LSRII flow cytometer using FACS DIVA software (Becton and Dickinson, NJ, USA); data were analyzed using FlowJo Software (Tree Star, OR, USA). Gating strategies are depicted in Figure S3.

### Quantification of T Cell Receptor Excision Circles (TRECs)

TRECs were quantified at baseline, 6, 12, 24, and 36 months of ART in 14 of the 33 patients (Figure S1). Quantification of sj-TRECs and DJβ1-TRECs was performed by nested polymerase chain reaction (PCR), using primers and standard curve's plasmids as previously described (25, 26). Briefly, 1 × 10^6^ PBMCs were lysed in Tween-20 (0.05%), nonidet P-40 (NP-40; 0.05 %), and Proteinase K (100 μg/mL) for 30 min at 56 °C followed by 15 min at 98 °C. Multiplex PCR amplification was performed using the specific “out” primers for sj-TREC or for each of the 6 DJβ1-TREC, together with the CD3 chain (10 min at 95 °C followed by 22 cycles of 30 s at 95 °C, 30 s at 60 °C and 3 min at 72 °C) using the 5′/3′ out primer pairs. Following the first round of amplification, each PCR product was 100-fold diluted; from the same diluted PCR product, TREC and CD3 were quantified in independent qPCR runs using a LightCycler® system (Roche Diagnostics, Basel, Switzerland). Using the specific “in” primers, qPCR amplification was as follows: 15 min at 95 °C, followed by 45 cycles of 1 s at 95 °C, 15 s at 55 °C and 10 s at 72 °C; fluorescence was measured at the end of each cycle. Results were expressed both as number of TRECs/10^5^ cells and number of TRECs/mL.

### Statistical Analysis

Statistical analysis was performed in the computing environment R (v3.4.2) using RStudio (v1.1.383). Overall, a *p*-value < 0.05 (α) was considered statistically significant; for multiple comparisons, Bonferroni corrections were applied and differences or effects that remained significant (<α/number of comparisons) are indicated in the Figures with a ^*^.

The R package *traj* (v1.2) was used to identify two clusters of patients based on their CD4^+^ T cell counts trajectories from baseline to 36 months of ART. The package implements the 3-step procedure proposed by Leffondré et al. (27) to identify clusters of longitudinal trajectories: first, 24 summary measures (Table S1) that describe features of the trajectories were calculated (28); second, a principal component analysis was performed on these 24 measures to select measures that best describe the main features of the trajectories, based on eigenvalues (>1); finally, trajectories were classified into clusters based on the previously selected factors.

We used the *nlme* package (v3.1.137) in R to fit nonlinear mixed effects models to the CD4^+^ T cell count and the CD4/CD8 ratio data, based on the exponential model described by Li et al. (9):

$$\begin{array}{l} X\left(t\right)=A+B\;\left[1-exp\left(-t\;/\;\tau \right)\right] \end{array}$$

where *X(t)* represents CD4^+^ T cell count or CD4/CD8 ratio at one given time point *t*; *A* represents the theoretical CD4^+^ T cell count or CD4/CD8 ratio at baseline; *B* represents the theoretical maximum increase that could be reached; *t* refers to time (in months) since ART initiation; and τ is a constant representing the timescale over which *X(t)* reaches its maximum value. The arguments used in the CD4^+^ T cell count models were: fixed = list (*A* ~ 1, *B* ~ group, τ ~ group) and random = *A* + *B* ~ 1 | Patient_ID, where Patient_ID correspond to the identification of each patient. The arguments used in the CD4/CD8 ratio models were: fixed = list (*A* ~ 1, *B* ~ group, τ ~1) and random = *A* + *B* + τ ~ 1 | Patient_ID.

Normality of data distributions was assessed by the Shapiro-Wilk test. Correlations were assessed using the Pearson or Spearman rank tests, depending on the underlying distribution. For quantitative data, depending on the underlying distributions, comparisons between two independent groups were performed using an independent *t*-test or Wilcoxon/Mann-Whitney *U*-test, unless otherwise stated. Cohen's *d* or *r*, respectively, was calculated as a measure of effect size. Within-group comparisons between two time points were performed with the paired-samples *t*-test (for approximately normally-distributed data); in this case, Cohen's *d* was calculated based on the standard deviation of the differences. For qualitative data, comparison was performed using Fisher exact test; effects sizes were described with the odds ratio (dichotomous variables) or Cramér's *V* (nominal variables with >2 categories) (29).

Effect sizes were assessed as follows:

- Cohen's *d*: small if <0.3; medium if [0.3,0.8]; or large if ≥0.8;
- *r*: small if <0.3; medium if [0.3, 0.5]; or large if ≥0.5;
- Cramér's V: small if <0.3; medium if [0.3,0.7]; or large if > 0.7;
- Correlation coefficient (*r* or ρ): negligible if <0.3; low if [0.3; 0.5]; moderate if [0.5; 0.7]; high if [0.7; 0.9]; or very high if [0.9; 1.0].

All statistical tests performed were two-tailed. Extreme outliers were defined as lower than P25 – 3 × Interquartile range (IQR) or higher than P75 + 3 × IQR. All tests were performed with and without extreme outliers to verify their effect on the *p*-value and all results pointed to the same direction (with significant or non-significant *p*-values) after exclusion of the extreme outliers. All the results reported included the extreme outliers.

Logistic regression analysis was conducted using the *glm()* function in R (v3.4.3) to identify associations between independent variables, assessed between baseline and 6 months on ART (inclusively), and the dependent variable “Being AIR or PIR” (Y = 1 for “AIR”; Y = 0 for “PIR”). Selection of variables for the multivariate analysis included a univariable logistic regression analysis for each candidate to detect potential predictors (*p*-value < 0.20). A backward stepwise selection procedure was performed, analyzing the significance of the coefficient for each variable at each step, until no further improvement was possible. The adequacy of the final model was assessed by means of a likelihood ratio test (LRT, reduced model vs. full model). The Akaike information criterion (AIC) was used to compare models with the same observations. The Hosmer-Lemeshow test for goodness-of-fit was applied to the final model and diagnostic plots were carried out as well, including ROC curve. The area under the curve (AUC) statistic was also calculated as a measure of goodness-of-fit, considering: [0.5; 0.7], poor; [0.7; 0.8], fair; [0.8; 0.9], good; and >0.9, excellent. For each model, all patients were classified as “probably PIR” (probability of being PIR ≥0.5) or “probably AIR” (probability of being PIR < 0.5). Sensitivity and specificity were calculated as the percentage of “probably PIR” among PIR and the percentage of “probably AIR” among AIR, respectively. Accuracy (ACC) was calculated as the percentage of patients PIR or AIR that were respectively classified as “probably PIR” or “probably AIR” among all patients. Relevant R code is available in Supplementary Material.

## Results

### Analysis of CD4^+^ T Cell Counts Trajectories Identified Two Clusters of Patients With Distinct Kinetics of Immune Recovery

Demographic and clinical characteristics of the patients are presented in Table 1.

**Table 1:** Demographic and clinical characteristics of HIV-infected patients included in the study.

	**Total *n* = 33**	**AIR *n* = 14**	**PIR *n* = 19**	**AIR vs. PIR**
**Gender**, male:female, *n* (%)	23:10 (70%: 30%)	9:5 (64%: 36%)	14:5 (74%: 26%)	*p* = 0.707^**a**^, OR = 0.64
**Age at baseline**, in years				
Mean ± SD Range [Min; Max]	42.2 ± 10.4 [23; 67]	38.1 ± 8.6 [23,51]	45.3 ± 10.8 [28; 67]	*t*_(31)_ = −2.032, *p* = 0.051^b^, Cohen's *d* = 0.72
**HIV viral load at baseline**, in log_10_ copies per mL, Median [Min; Max]	5.57 [4.78; 6.98]	5.96 [5.25; 6.98]	5.41 [4.78; 6.84]	***U*** **=** **203**, ***p*** **=** **0.011**^**c**^, ***r*** **=** **−0.44**
**CD4**^**+**^ **T cell count at baseline**, in cells/μL, Median [Min; Max]	67 [8; 193]	93 [12; 193]	61 [8; 182]	*U* = 167, *p* = 0.229^**c**^, *r* = −0.21
**CD4/CD8 ratio at baseline**,				
Mean ±*SD* Range [Min; Max]	0.122 ± 0.095 [0.010; 0.373]	0.160 ± 0.113 [0.017; 0.373]	0.095 ± 0.070 [0.010; 0.272]	*t*_(31)_ = 2.032, *p* = 0.051^b^, Cohen's *d* = 0.72
Clinical categories^*^, *n* (%)				
A (Asymptomatic or acute HIV infection)	3 (9%)	1 (7%)	2 (11%)	
B (not A or C)	14 (42%)	4 (29%)	10 (53%)	*p* = 0.262^**a**^, Cramér's V = 0.27
C (AIDS-defining conditions)	16 (48%)	9 (64%)	7 (37%)	
**Co-infections at baseline**, *n* (%)				
HCV	9 (27%)	2 (14%)	7 (37%)	*p* = 0.241^**a**^, OR = 3.50
HBV	1 (3%)	1 (7%)	0 (0%)	*p* = 0.424^**a**^, OR = 0.00
**HIV subtype**, *n* (%)				
B	13 (39%)	5 (36%)	8 (42%)	
G	11 (33%)	4 (29%)	7 (37%)	
C	4 (12%)	2 (14%)	2 (11%)	*p* = 0.633^**a**^, Cramér's V = 0.40
Other	4 (12%)	3 (21%)	1 (5%)	
Unknown	1 (3%)	0 (0%)	1 (5%)	
**HIV transmission mode**, *n* (%)				
Intravenous drug user	6 (18%)	1 (7%)	5 (26%)	
Men who have sex with men	9 (27%)	5 (36%)	4 (21%)	
Heterosexual	15 (45%)	6 (43%)	9 (47%)	*p* = 0.567^**a**^, Cramér's V = 0.27
Other	2 (6%)	1 (7%)	1 (5%)	
Unknown	1 (3%)	1 (7%)	0 (0%)	
**Time from diagnosis to baseline**, in months, Median [Min; Max]	5 [0; 128]	23 [1; 85]	3 [0; 128]	*U* = 179, *p* = 0.096^**c**^, *r* = −0.29
**ART regimen components**, *n* (%)				
2 NRTIs: TDF^+^FTC	29 (88%)	14 (100%)	15 (79%)	
ABC^+^3TC	3 (9%)	0 (0%)	3 (16%)	*p* = 0.244^**a**^, Cramér's V = 0.32
AZT^+^3TC	1 (3%)	0 (0%)	1 (5%)	
3^rd^ Drug: EFV	25 (76%)	10 (71%)	15 (79%)	
DRV/r	6 (18%)	4 (29%)	2 (11%)	*p* = 0.473^**a**^, Cramér's V = 0.30
LPV/r	1 (3%)	0 (0%)	1 (5%)	
NVP	1 (3%)	0 (0%)	1 (5%)	
**HIV drug resistance**, *n* (%)				
Yes	11 (33%)	3 (21%)	8 (42%)	
No	21 (64%)	11 (79%)	10 (53%)	*p* = 0.266^**a**^, OR = 0.34
Unknown	1 (3%)	0 (0%)	1 (5%)	

* *Based on Centers for Disease Control and Prevention (CDC) Classification System*.

a *Fisher's exact test*.

b *Independent t-test*.

c *Wilcoxon/Mann-Whitney U test*.

*Values in bold indicate statistically significant difference (p < 0.05) between AIR and PIR. ABC, abacavir; DRV/r, ritonavir boosted darunavir; EFV, efavirenz; FTC, emtricitabine; HBV, Hepatitis B virus; HCV, Hepatitis C virus; LPV/r, ritonavir boosted lopinavir; NRTI, nucleoside or nucleotide analogue reverse transcriptase inhibitors; NVP, nevirapine; OR: odds ratio; TDF, tenofovir disoproxil fumarate; 3TC, lamivudine*.

**Figure 1:**
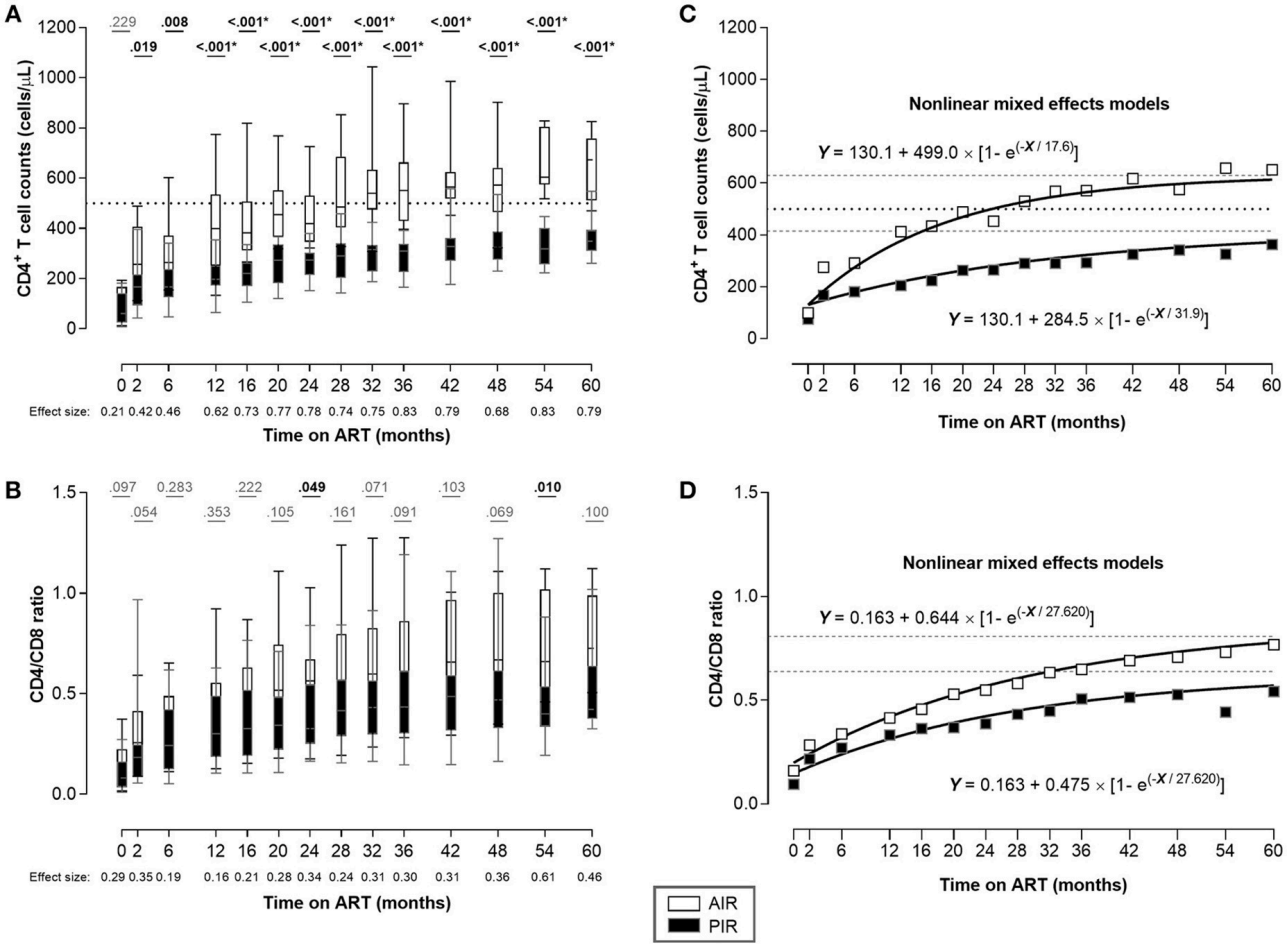
Despite having similar CD4^+^ T cell counts at ART onset, AIR reached higher CD4^+^ T cell counts as soon as 2 months after ART initiation. **(A)** CD4^+^ T cell counts and **(B)** CD4/CD8 ratio were compared throughout ART between AIR and PIR. A Wilcoxon-Mann-Whitney U-test was performed to compare the two groups of patients at each time point, and the correspondent r is presented as a measure of effect size. *Significant after Bonferroni correction (α/14 = 0.004). **(C**,**D)** Nonlinear mixed effects models for CD4^+^ T cell count and CD4/CD8 ratio evolutions, respectively. White squares represent AIR mean of CD4^+^ T cell count **(C)**, or CD4/CD8 ratio **(D)** at each time point; black squares represent PIR mean of CD4^+^ T cell count **(C)**, or CD4/CD8 ratio **(D)** at each time point. Black dotted lines in **(A)** and **(C)** correspond to Y = 500. Gray dashed lines correspond to the asymptotes: Y = 629.1 and Y = 414.6 **(C)**; Y = 0.807 and Y = 0.638 **(D)**, respectively.

The CD4^+^ T cell count trajectories of the 33 patients were automatically analyzed using R *traj* package, and two clusters were formed (Figure 1A; Table S2). From the summary measures considered for each trajectory (Table S1), the main descriptors of the CD4^+^ T cell count trajectories were range and change relative to the mean over time.

All patients in cluster 1 (*n* = 14) had at least one CD4^+^ T cell count above 500 cells/μL during the first 36 months of ART, and were classified as AIR. Patients in cluster 2 (*n* = 19) had all CD4^+^ T cell counts bellow 500 cells/μL, during the same time period, and were classified as PIR (Figures 1A, S4A,B).

At baseline, PIR tended to be older, with lower CD4/CD8 ratios, and had significantly lower plasma viral loads; there were no significant differences in baseline CD4^+^ cell counts between PIR and AIR (Table 1). After 12 months of ART, AIR exhibited significantly higher CD4^+^ T cell counts than PIR. This difference became progressively more evident throughout follow-up (Figure 1A). AIR consistently presented higher median rates of increase of CD4^+^ T cell counts during the first 12 months of ART (Table 2). The CD4/CD8 ratio tended to be lower in PIR than AIR at all time points (Figures 1B, S4C,D).

**Table 2:** CD4^+^ T cell count slopes during ART.

	**CD4^+^ T cell count slope**, in cells/μL/month^a^, Median [P25; P75]			
**Time interval during ART, in months**	**Total n = 33**	**AIR n = 14**	**PIR n = 19**	**AIR vs. PIR**
[0; 2]	57 [29; 102]	87 [50; 107]	48 [21; 69]	**U = 191, p = 0.036**^b^, **r = 0.36**
[2,12]	7 [2,13]	14 [10,17]	3 [1,7]	**U = 228**, **p < 0.001**^b^, **r = 0.60**
[12,24]	5 [2,10]	6 [-1; 13]	5 [3,10]	U = 135, p = 0.956^b^, r = 0.01
[24,36]	3 [1,9]	12 [2,17]	2 [1,4]	**U = 198**, **p = 0.019**^b^, **r = 0.41**
[36,48]	3 [0; 8]	2 [−4; 10]	3 [1,7]	U = 68, p = 0.544^b^, r = 0.12
[48,60]	5 [1,9]	7 [1,11]	5 [1,7]	U = 23, p = 0.830^b^, r = 0.06

a *CD4 count slopes from time point t_i_ to t_f_ were calculated for each individual by the least squares estimation method, including all the available CD4 counts between t_i_ and t_f_*.

b *Wilcoxon/Mann–Whitney U-test*.

*Bold values indicate statistically significant difference (p < 0.05) between AIR and PIR*.

The adjusted kinetics for CD4^+^ T cell counts and CD4/CD8 ratio in PIR, obtained using nonlinear mixed effects models, were:

$$\begin{array}{l} \text{CD}4\text{ count }\left(t\right)=\;130.1\;+\;284.5\;\left[1\;-\;exp\left(-t/31.9\right)\;\right]\\ \text{CD}4/\text{CD}8\;\left(t\right)=0.163\;+\;0.475\;\left[1-exp\left(-t/27.620\right)\right] \end{array}$$

and in AIR:

$$\begin{array}{l} \text{CD}4\text{ count }\left(t\right)=\;130.1\;+\;499.0\;\left[1\;-\;exp\left(-t/17.6\right)\;\right]\\ \text{CD}4/\text{CD}8\;\left(t\right)=0.163\;+\;0.644\;\left[1-exp\left(-t/27.620\right)\right] \end{array}$$

These kinetics indicate that on long-term ART, mean CD4^+^ T cell counts among PIR asymptoted to 414.6 cells/μL and the CD4/CD8 ratio to 0.638; these quantities stabilized at 629.1 and 0.807, respectively, in AIR (Figures 1C,D).

### CD4^+^ T Cell Proliferation and Activation Status Did Not Differ Between PIR and AIR

**Figure 2:**
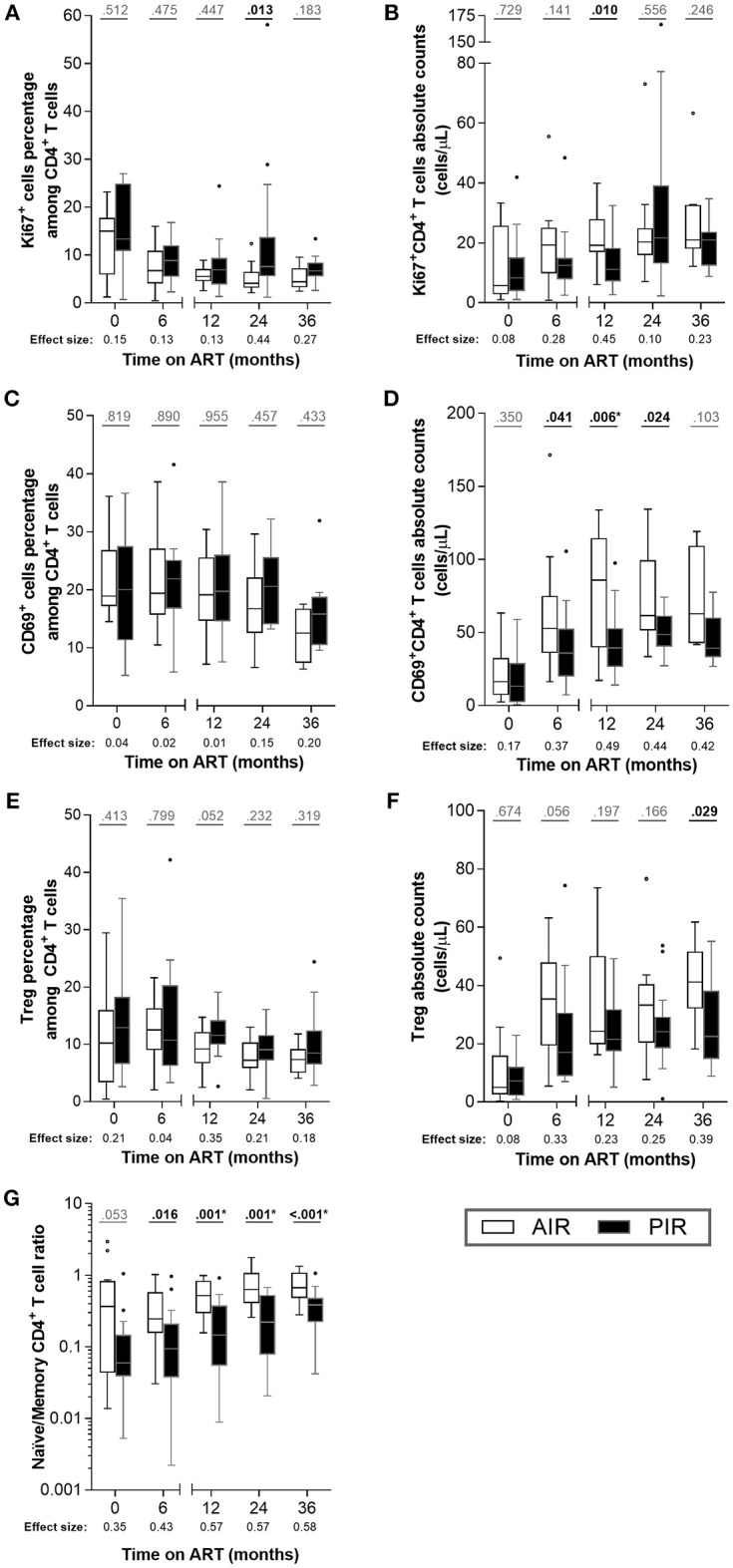
Throughout ART, PIR and AIR presented no major differences in CD4^+^ T cell activation status, proliferation, Treg numbers, nor naïve/memory CD4^+^ T cell ratio. **(A)** The percentage among CD4^+^ T cells and **(B)** absolute number of Ki67^+^ cells, **(C,D)** CD69^+^ cells, **(E,F)** Treg (CD25^high^CD127^−^FoxP3^+^CD4^+^ T cells) and **(G)** naïve/memory CD4^+^ T cell ratio were compared throughout therapy in AIR and PIR. A Wilcoxon-Mann-Whitney *U*-test was performed to compare the two groups of patients at each time point, and the corresponding *r* is presented as a measure of effect size. *Significant after Bonferroni correction (α/5 = 0.010).

We did not detect differences in CD4^+^ T cell division rates, as assessed by Ki67 expression (Figures 2A,B, S5A,B), nor in their activation status (CD69^+^ cells; Figure 2C). Absolute numbers of CD69^+^CD4^+^ T cells were higher in AIR at 12 months of ART, reflecting their greater CD4^+^ T cell counts (Figures 2D, S5C,D).

Diverse immune recoveries have been associated with differences in CD4^+^ T cell proliferation and activation (30–32). To assess whether the apparent discrepancy between these associations and our results could be related to CD4^+^ T cell counts at baseline, we analyzed a group of HIV-infected patients who started ART with CD4^+^ T cell counts in the range 200–350 cells/μL, who achieved full virologic suppression and adequate immune recovery (referred to as HIV-infected_BL[200−350]_). These individuals presented lower percentages of Ki67^+^ cells and of CD69^+^ cells among CD4^+^ T cells than PIR or AIR at baseline (Figure S7).

No substantial differences were observed between AIR and PIR in either percentage or absolute numbers of Treg (CD25^high^CD127^−^Foxp3^+^ CD4^+^ T cells; Figures 2E,F, S5E,F).

The naïve/memory CD4^+^ T cell ratio (i.e., CD45RA^+^CD45RO^−^/CD45RA^−^CD45RO^+^) was tendentially higher in AIR than in PIR at baseline and 6 months, and significantly higher at 12, 24, and 36 months of ART (Figures 2G, S4E,F), which is consistent with naïve cells contributing more to CD4^+^ T cell recovery in AIR than in PIR.

### AIR Presented Higher Thymic Function Since Baseline, Independently of Age Differences

**Figure 3:**
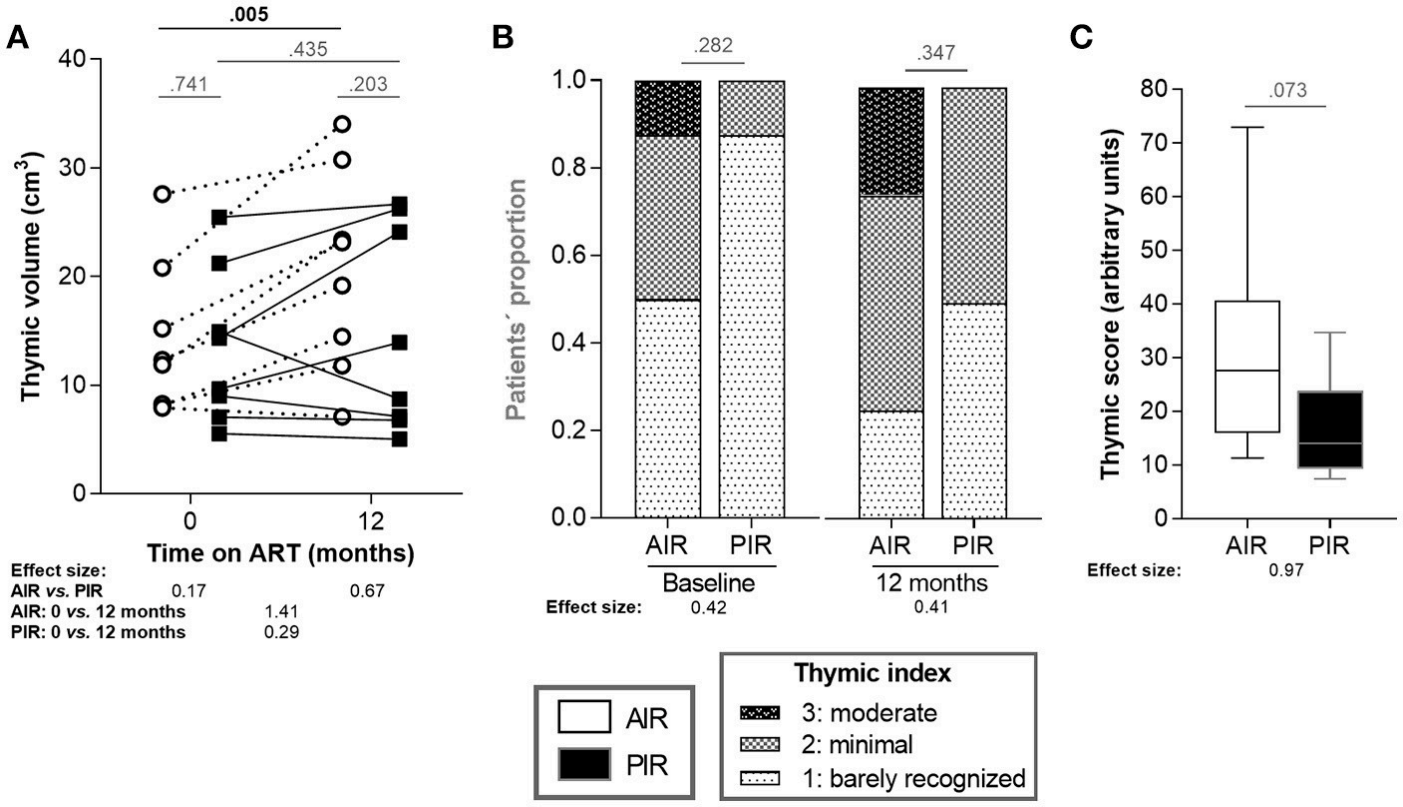
AIR presented thymic volume increase during the first 12 months of ART. **(A)** Thymic volumes and **(B)** thymic indices were compared between AIR and PIR at baseline and at 12 months of ART. Thymic indices ranged from 1 to 3. **(C)** Thymic scores were calculated as the product of thymic volume and index means, over the first year of ART. Thymic volumes and scores **(A,C)** were compared using independent *t*-tests and the correspondent effect size estimates were calculated using for Cohen's *d*. Thymic indices **(B)** were compared using Fisher's exact test and effect size estimates were calculated using Cramér's *V*.

Thymic function was characterized using three distinct metrics: (1) assessment of thymic index (defined in the imaging subsection of the Methods) and volume, at baseline and 12 months of ART; (2) quantification of peripheral blood TRECs at baseline, 6, 12, 24, and 36 months of ART); and (3) quantification of recent thymic emigrants (RTE; CD4^+^CD3^+^CD45RA^+^CD31^+^) in blood, at baseline, 6, 12, 24, and 36 months of ART. AIR and PIR presented similar thymic volumes at baseline and at 12 months of ART. However, AIR exhibited significant increases in thymic volume during that period, while PIR did not (variations of 6.47 ± 4.59 cm^3^ vs. 1.43 ± 4.89 cm^3^, respectively; Figure 3A). PIR tended to present lower thymic index, both at baseline and 12 months of ART (Figure 3B). A thymic score was calculated as the product of thymic volume and index means, over the first year of ART. AIR had a tendency to present higher thymic scores (Figure 3C). We also compared thymic volume, index and score between HIV-infected_BL[200−350]_ and AIR and PIR, respectively. Interestingly, AIR, but not PIR, presented higher thymic volume at 12 months of ART and higher thymic score than HIV-infected_BL[200−350]_ (Figure S8).

**Figure 4:**
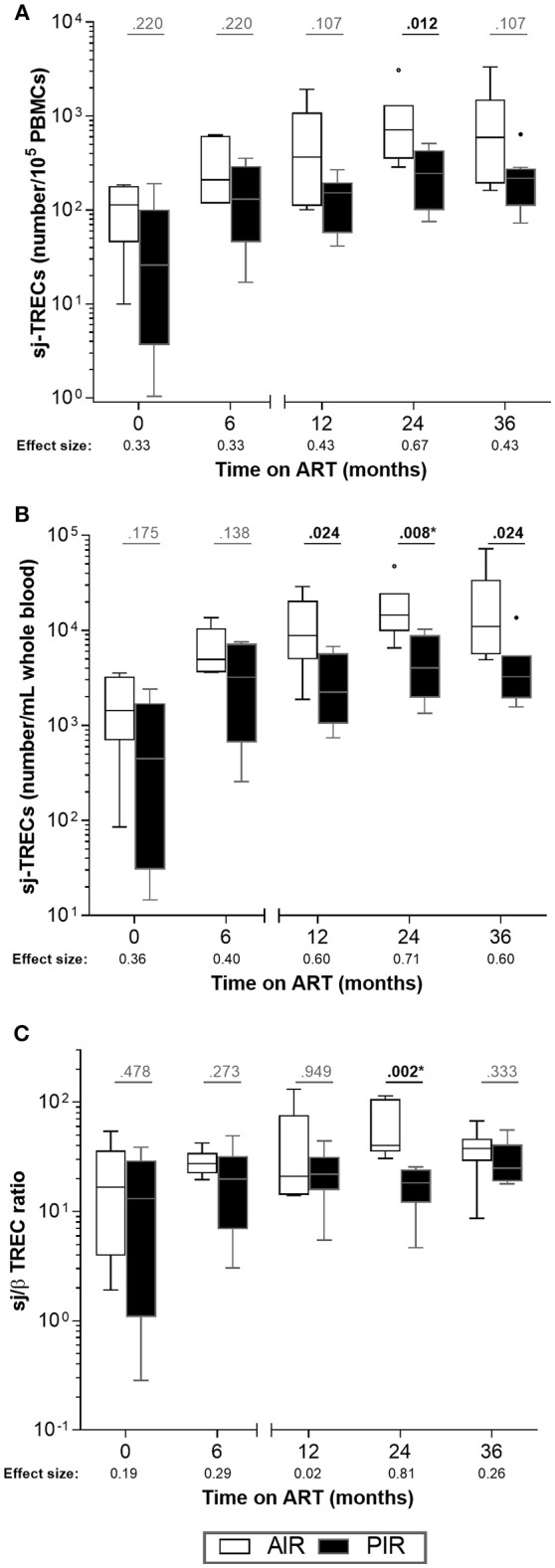
No major differences were observed in intrathymic proliferation between AIR and PIR. **(A)** sj-TRECs/10^5^ PBMCs, **(B)** sj-TRECs/mL of whole blood and **(C)** sj/β TREC ratios were compared throughout ART between AIR and PIR. A Wilcoxon-Mann-Whitney *U*-test was performed to compare the two groups of patients at each time point, and the corresponding *r* is presented as a measure of effect size. *Significant after Bonferroni correction (α/5 = 0.010).

**Figure 5:**
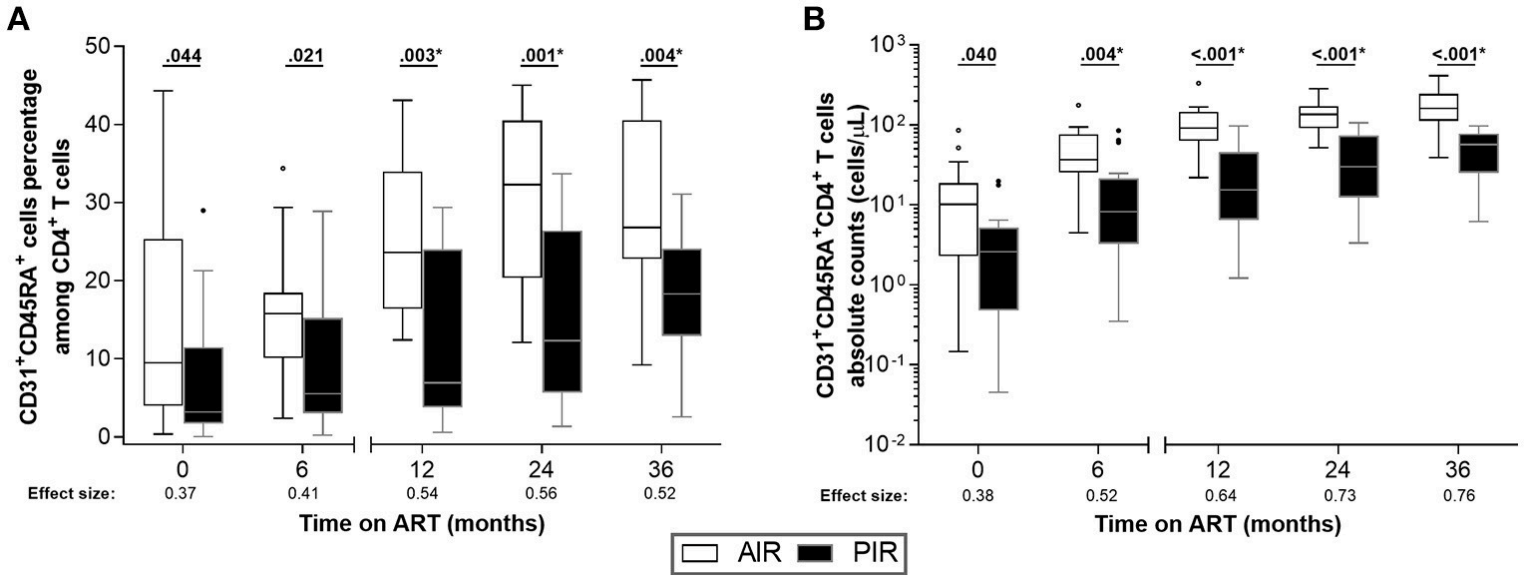
AIR had higher percentages of RTE (CD31^+^CD45RA^+^) among CD4^+^ T cells since baseline. **(A)** RTE percentage among CD4^+^ T cells and **(B)** their absolute numbers were compared throughout ART between AIR and PIR. A Wilcoxon-Mann-Whitney *U*-test was performed to compare the two groups of patients at each time point, and the correspondent r is presented as a measure of effect size. *Significant after Bonferroni correction (α/5 = 0.010).

Furthermore, AIR tended to present higher frequencies of sj-TRECs (numbers per 10^5^ PBMCs) at 24 months of ART (Figure 4A), and significantly higher numbers of sj-TRECs/mL of blood and sj/β TREC ratios at the same time point (Figures 4B,C, S6A,B; Table S3). Absolute numbers and percentage of RTE among CD4^+^ T cells were higher in AIR than in PIR throughout the follow-up period, except at baseline and, for the percentage of RTE, at 6 months of ART (Figures 5A,B, S6C–F; Table S4).

We found no correlation between patients' age and any of the surrogates of thymic function (Table S5). We also found no correlations between thymic volume (at baseline or at 12 months of ART) and RTE absolute number or percentage among CD4^+^ T cells at any other time point.

### Age, HIV Viral Load, and RTE Percentages at Early Stages of ART Had Predictive Value for Immune Recovery, in Addition to CD4^+^ T Cell Counts

We performed univariate logistic regressions with all variables to identify predictors of PIR or AIR status. The variables that performed the best (considering the lowest LRT *p*-values) were: CD4^+^ T cell count at 6 months of ART (LRT *p*-value = 0.003, AUC = 0.774, ACC = 69.7%), CD4^+^ T cell count at 2 months of ART (LRT *p*-value = 0.007, AUC = 0.750, ACC = 74.2%) or CD4^+^ T cell count slope from baseline to 6 months of ART (LRT *p*-value = 0.010, AUC = 0.756, ACC = 60.6%). Performing combinations of eight elements from the set of variables identified in the univariate analysis with *p* < 0.200, and applying a stepwise selection, four logistic models, with three independent variables and an area under the curve (AUC) >0.80, were obtained (Models 1–4). Each of the models were represented by the following equation:

$$\begin{array}{l} \begin{array}{c} \text{Probability}\\ \text{of being}\\ \text{PIR} \end{array}=\frac{1}{1+e^{\left(a\;+\;b\;\times \text{ Predictor }1\;+\;c\;\times \text{ Predictor }2\;+\;d\;\times \text{ Predictor }3\right)}} \end{array}$$

replacing *a, b, c, d* and the predictors by the coefficients and respective variables listed in Table 3. Predictors included the following variables in different combinations: age at baseline; viral load at baseline; RTE percentage among CD4^+^ T cells at 6 months of ART; CD4^+^ T cell count slope from baseline to 6 months of ART; ratio of RTE percentages among CD4^+^ T cells at baseline and 6 months of ART; CD4^+^ T cell counts at 2 and 6 months of ART. The best of these four models regarding the goodness-of-fit (AUC = 0.91) and accuracy (87.1%), combined age, plasma viral load at baseline and CD4^+^ T cell counts at 2 months of ART (Table 3, Model 4). To compare the relative quality of the models using AIC, we used a fixed data set with no missing values. Data of five patients had to be excluded, and four models (Model 1′ to 4′), including the same variables of Models 1–4, were built using data from the remaining 28 patients. Considering the AIC, the best of these models combined age, plasma viral load at baseline and CD4^+^ T cell counts at 6 months of ART (Table S6, Model 3′).

**Table 3:** Statistical comparison of the multivariate logistic regression models.

**Logistic regression**							
**Models**	**Coefficients**	**Predictor variables**	**LRT**^**a**^	***p*****-value**^**b**^	**ROC analysis**		
					**AUC**	**Accuracy**^**c**^	**Sensitivity/ Specificity**
Model 1 (*n* = 31)	*a* = −8.670 *b* = −0.102 *c* = 1.985 *d* = 0.105	Age at baseline Log HIV plasma VL at baseline % RTE among CD4^+^ T cells at 6 mo	*p* = 0.537	0.084 0.019 0.061	0.861	77.4%	76.5/78.6%
Model 2 (*n* = 30)	*a* = −15.182 *b* = 2.519 *c* = 0.133 *d* = −2.179	Log HIV plasma VL at baseline CD4^+^ T cell count slope [0, 6] mo % RTE among CD4^+^ T cells ratio _(0/6mo)_	*p* = 0.112	0.051 0.029 0.038	0.871	76.7%	81.3/71.4%
Model 3 (*n* = 33)	*a* = −9.703 *b* = −0.103 *c* = 1.897 *d* = 0.013	Age at baseline Log HIV plasma VL at baseline CD4^+^ T cell count at 6 mo	*p* = 0.885^*^	0.071 0.029 0.037	0.902	84.8%	89.5/78.6%
Model 4 (*n* = 31)	*a* = −10.971 *b* = −0.160 *c* = 2.610 *d* = 0.012	Age at baseline Log HIV plasma VL at baseline CD4^+^ T cell count at 2 mo	*p* = 0.333^†^	0.038 0.018 0.017	0.908	87.1%	94.1/78.6%

*Models are ordered by increasing sensitivity*.

a *Likelihood ratio test (LRT) was used to compare the goodness of fit of reduced model (three final variables) vs. full model (initial variables); a non-significant p-value means that the reduced model is as good as the full model*.

b *p-Value for each of the three predictors of the reduced model*.

c *Accuracy corresponds to the percentage of patients PIR or AIR that were respectively classified as “probably PIR” or “probably AIR” among all patients*.

* *LRT was performed using only 31 of the 33 patients due to missing values in variables that were excluded during the stepwise selection*.

† *LRT was performed using only 29 of the 31 patients due to missing values in variables that were excluded during stepwise selection*.

*AUC, area under the ROC curve; LRT, likelihood ratio test; mo, months; p, p-value; ROC, receiver operating characteristic curve; VL, viral load*.

For each of the Models 1–4, patients that failed to be correctly identified (i.e., PIR identified as “Probably AIR” or AIR identified as “Probably PIR”) presented values in the included variables that, in combination, led to their inclusion in the opposite patient group (Table S7). We found no single variable responsible for the failure of the model in predicting patient's immune recovery outcome.

## Discussion

Despite the extensive literature regarding PIR, there is currently no consensus on their definition, hampering their identification and clinical management and contributing to their poorer prognoses compared to other HIV-infected patients. Here, we aimed to distinguish and define PIR and AIR by clustering analysis of the trajectories of CD4^+^ T cell counts during therapy, and to build logistic models to predict PIR status using parameters assessed during the first months of ART. Although mathematical tools have been previously used to separate PIR and AIR [e.g., Pérez-Santiago et al. used random forest classification and unsupervised clustering to explore different definitions of a discordant immune response (33)], to our knowledge, the use of longitudinal cluster analysis has not been reported in this context. Our results revealed that PIR presented lower HIV viral load, CD4/CD8 ratio, naïve/memory CD4^+^ T cell ratio and, most importantly, lower thymic function, strengthening the importance of thymus in immune recovery during ART. Furthermore, our logistic models were able to correctly predict PIR/AIR outcome after 36 months of therapy in up to 87% of cases, based on observations made until 2–6 months after ART onset.

This study included only severely lymphopenic patients (<200 CD4^+^ T cells/μL), in order to limit the effect of variation in counts at baseline, which itself can explain distinct patterns of immune recovery (34). Limiting our sample to severely lymphopenic patients is also of particular interest given that these patients present a higher risk of new AIDS-defining events and death (22, 35).

The importance of the thymus in HIV pathogenesis and in immune recovery during ART is well-recognized. Several studies have shown that both adults and children infected by HIV present thymic alterations that may be reversed during ART; and that higher estimates of thymic function in patients on therapy are associated with higher CD4^+^ T cell counts and better immune recoveries (36–38). Here we saw that AIR exhibited higher levels of thymic function than PIR by the following metrics: higher percentage of RTE among CD4^+^ T cells at 12, 24, and 36 months of ART; higher number of sj-TRECs and a greater sj/β TREC ratio at 24 months of ART; and significant increase in thymic volume between baseline and 12 months of ART. The observation regarding RTE percentages suggests that thymic function is superior in AIR before and during therapy, and thymic volume gain in AIR, but not in PIR, points to a higher functional regeneration potential/capacity. Interestingly, our data also suggest that thymic function regeneration is more evident in AIR in comparison to a group of HIV-infected individuals initiating ART with CD4^+^ T cell counts ranging between 200 and 350 cells/μL. The observed differences in naïve/memory CD4^+^ T cell ratio and RTE percentage among CD4^+^ T cells are particularly interesting as they may relate to the mechanism behind the divergent trajectories of immune recovery between PIR and AIR. They point to a scenario in which the two groups present different dynamics within the naïve and memory CD4^+^ T cell subpopulations, probably related to distinct thymic functions. These results are in accordance with previous studies focusing on poor immunologic responses to ART, either with case-control (39), cross-sectional (40) or longitudinal designs (21, 41).

RTE were identified as CD31^+^CD45RA^+^CD4^+^ T cells, which represent a subset of naïve CD4^+^ T cells with high TREC content (42). Some limitations in the usage of CD31 have been identified, such as: (1) TCR-induced activation down-regulates CD31 expression (43, 44); (2) some CD4^+^CD45RA^+^CD31^+^ cells undergo *in vivo* peripheral proliferation without immediate loss of CD31, resulting in an accumulation of CD45RA^+^CD31^+^ proliferative offspring (45, 46); (3) thymectomised individuals maintain a population of CD31^+^ T cells in circulation (47). Notwithstanding these limitations, it is still one of the most well established markers of RTE.

A decrease in thymic function is associated with aging (48–50). Even though we found no significant age differences between PIR and AIR, PIR tended to be older, a tendency that might explain the reduced thymic function presented by this group. This possibility is particularly important given that age at baseline was included in the predictive models discussed below. If age alone could account for the differences in surrogates of thymic function, we would expect an inverse correlation between age and these parameters. Such correlations have been described by others in the context of HIV infection or hematopoietic stem-cell transplantation (16, 51, 52). However, we saw no correlations between age and any surrogates of thymic activity in our cohort, which might be related to the narrow age range of this cohort: all but two AIR and one PIR were over age 30. It might also be that improvement in thymic function during ART is, to a certain extent, independent of patient age, at least for these patients' age range.

PIR presented significantly lower HIV plasma loads at baseline, as reported in other studies (53–56). This association may seem counterintuitive, given that higher plasma viral loads have been associated with disease progression and poorer prognosis (57–59). One possible explanation is that the high HIV plasma loads in AIR are the main factor contributing to the lymphopenia, so the suppression of HIV replication by ART results in an adequate immunological response; while in PIR, typically with low HIV plasma loads, different mechanisms contribute to the lymphopenia and the effectiveness of ART to counterbalance each of these mechanisms is limited.

To build predictive models to identify PIR as early as possible, we focused on parameters at baseline, 2 or 6 months of ART. While the univariate logistic regressions showed that CD4^+^ T cell counts at 2 or 6 months of ART, and the slope between baseline and 6 months of ART were the single variables with highest predictive value, the multivariate analyses allowed us to build models with greater prediction accuracies. Of the four multivariate models described here, the one including age and plasma viral load at baseline, and RTE percentage among CD4^+^ T cells at 6 months of ART (Model 1) performed better than any of the univariate models, despite excluding CD4^+^ T cell counts. Furthermore, the model which included age and plasma viral load at baseline, and CD4^+^ T cell count at 2 months of ART (Model 4) was able to correctly predict AIR/PIR status in 87.1% of the cases, with high sensitivity and specificity (94.1 and 78.6%, respectively). Although these results are encouraging, it should be highlighted that they were obtained using a relatively small number of patients of a single hospital. Therefore, replication of our analyses in other cohorts or encompassing larger numbers of patients will be of great value.

In contrast with our results, others have shown greater numbers of Treg and higher levels of cell proliferation and activation in PIR (30–32). These differences might be due to the exclusive analysis of patients with CD4^+^ T cell counts <200 cells/μL at ART onset in our study, or to distinct criteria to define PIR and AIR. It has been shown that severely lymphopenic patients present higher percentage of Treg, lower percentage of Treg with a naïve phenotype and impaired production of Treg by the thymus, a phenotype not observed for patients starting ART with higher CD4^+^ T cell counts (>350 cells/μL) (60).

The major strengths of this study are the longitudinal prospective design, with several evaluations over at least 36 months of therapy, and the multi-parametric evaluation of thymic activity, since each method alone is indirect and presents its own advantages and limitations [reviewed in (61)]. Our results show that persistent differences in thymic function in severely lymphopenic HIV-infected patients are associated with distinct immune recoveries that diverge soon after ART initiation. The outcome of PIR can be improved by early identification of these patients. This will prompt closer follow-up and possibly, in the future, the administration of strategies to boost thymic function. As pre-clinical and clinical trials aiming to enhance thymic function are currently on-going, studies like the one presented here might help to select the patients that could benefit the most from novel therapeutic approaches.

## Ethics Statement

Peripheral blood samples were provided by patients with chronic HIV-1 infection, who participated in the prospective cohort study authorized by the local ethics committee (reference 168/CES). From all participating patients, an informed written consent was obtained.

## Author Contributions

RR-S, AH, and MC-N conceptualized the study. CN, AH, and MC-N designed experiments. RR-S, CN, IF, JC-G, and AH performed experiments. RR-S, CA, and EA performed statistical analysis. RR-S and CN prepared the figures. RC, AY, and MC-N supervised research. All authors discussed the results and contributed to the final manuscript.

### Conflict of Interest Statement

AH is a consultant for Abbvie LDA, Bristol-Myers Squibb, Gilead Sciences, Janssen-Cilag, Merck Sharp and Dohme and ViiV Healthcare. The remaining authors declare that the research was conducted in the absence of any commercial or financial relationships that could be construed as a potential conflict of interest.

## Abbreviations

ACC: accuracy
AIC: Akaike information criterion
AIR: adequate immunological responders
ART: antiretroviral therapy
AUC: area under the curve
CT: computed tomography
IQR: interquartile range
LRT: likelihood ratio test
PBMCs: peripheral blood mononuclear cells
PIR: poor immunological responders
RTE: recent thymic emigrants
TRECs: T cell receptor excision circles
Treg: regulatory T cells.
