# Supplementary material for: Thymic Function as a Predictor of Immune Recovery in Chronically HIV-Infected Patients Initiating Antiretroviral Therapy
Source: Front Immunol. 2019 Feb 5;10:25. doi: 10.3389/fimmu.2019.00025 (PMC6370619; doi:10.3389/fimmu.2019.00025)
Supplement: Supplementary file 1 [file Data_Sheet_1.docx]

Supplementary material

I - SUPPLEMENTARY statistical analysis information: Relevant R code

II - SUPPLEMENTAry Tables

- Table S1. Measures computed in the first step of the clustering analysis.
- Table S2. Clusters of CD4^+^ T cell count trajectories and corresponding subsets of patients.
- Table S3. Signal joint TRECs and sj/β TREC ratio.
- Table S4. Absolute number and percentage of CD4^+^CD45RA^+^CD31^+^ T cells in peripheral blood.
- Table S5. Correlations between age and thymic function surrogates.
- Table S6. Statistical comparison of multivariate logistic regression models.
- TableS s7. “Probability of being PIR” among AIR and PIR, according to each multivariate logistic regression model.

III - Supplementary Figures

- Figure S1. Diagram of patient selection and clustering.
- Figure S2. Viral load throughout the follow-up period.
- Figure S3. Flow Cytometry gating strategies.
- FIGURE S4-6. Spaghetti plots depicting data evolution for each patient, throughout antiretroviral therapy.
- FIGURES S7-8. Comparison between patients with CD4^+^ T cell count of [200; 350] and patients with <200 cells/µL at baseline, throughout antiretroviral therapy.

relevant R code

part 1: Longitudinal cluster analysis

# Import data

library(xlsx, quietly = TRUE)

dfxt <-read.table("CD4_counts_0-36mo.txt",header=T) # The txt file contains CD4+ T cell counts of the 33 patients from baseline to 36 months of ART

Time <-data.frame(Patient_ID=dfxt$Patient_ID,time.1=rep(0,33),time.2=rep(2,33),time.3=rep(6,33),time.4=rep(9,33),time.5=rep(12,33),time.6=rep(16,33),time.7=rep(20,33),time.8=rep(24,33),time.9=rep(28,33),time.10=rep(32,33),time.11=rep(36,33))

Data<-dfxt

dim(Data)

dim(Time)

LstData<-list(CD4=Data,TimePoints=Time)

head(LstData$CD4)

head(LstData$TimePoints)

Data=LstData$CD4

Time=LstData$TimePoints

# Run step1 (measures), step2 (factors) and step3 (clusters)

s1 = step1measures(Data,Time, ID=TRUE)

s2 = step2factors(s1)

s3 = step3clusters(s2, nclusters = 2)

# Print and plot "traj object"

s3

plot(s3)

summary(s3)

# Plot mean combination trajectories

plotCombTraj(s3)

# Plot mean trajectories

plotMeanTraj(s3)

# Plot median trajectories

plotMedTraj(s3)

# Display measures

head(s1$measurments)

# Plot mean trajectory of all individuals

plot(s1$measurments$ID, s1$measurments$m5)

# Display factors

head(s2$factors)

# Display "traj object”

plot(s3)

s3$cluster

f <- as.factor(s3$cluster[,2])

table(f)

s3$cluster[which(s3$cluster[,2]==1),1]

s3$cluster[which(s3$cluster[,2]==2),1]

part 2: Logistic regression models

# Import data

library(xlsx, quietly = TRUE)

df <- read.xlsx2("File1.xlsx", sheetIndex = 1, stringsAsFactors = FALSE) # File 1 contains all clinical and laboratorial data

df[df == "NA"] <- NA

### Univariate logistic regression analysis

Model.1u<-glm(groups ~ V1, data = df, family = binomial()) # V1, Variable 1

# P(“Being PIR”) = 1 / [1+exp(1)^ (-(b0+b1*V1)) ]

summary(Model.1u)

# Chi-square:

modelChi <- Model.1u$null.deviance - Model.1u$deviance # deviance for the model

chidf <- Model.1u$df.null - Model.1u$df.residual # degrees of freedom

chisq.prob <- 1 - pchisq(modelChi, chidf)

### Multivariate logistic regression analysis

# Example: Model 1

Model.1m <- glm(groups ~. , data = dfx1, family=binomial)

full.model <- Model.1m # 8 variables: V1 to V8

stepAIC(Model.1m) # V4, V5 and V8 out

redAIC <- glm(subg~V1 + V2 + V3 + V6 + V7,

data = dfx1, family=binomial)

summary(redAIC) # V7 out

redAIC.2 <- glm(subg~V1 + V2 + V3 + V6,

data = dfx1, family=binomial)

summary(redAIC.2) # V6 out

redAIC.3 <- glm(subg~V1 + V2 + V3,

data = dfx1, family=binomial)

summary(redAIC.3)

reduced.model <- redAIC.3 # 3 variables

# Likelihood ratio test

anova(redAIC.3, Model.1m, test="Chisq") # p-value > 0.05

# Akaike information criterion

summary(reduced.model)

# Hosmer–Lemeshow test

library(MKmisc)

HLgof.test(fit=fitted(reduced.model),obs=dfx2)

# ROC analysis

library(epiDisplay)

lroc(reduced.model)

lroc(reduced.model)$auc

# Sensitivity (pa.PIR), specificity (pa.AIR) and accuracy (pa.total)

pa.PIR <- sum(df.md1[which(df.md1$groups==”PIR”),"Probability_Model.1"]>0.5)/n1

pa.AIR <- sum(df.md1[which(df.md1$groups==”AIR”),"Probability_Model.1"]<0.5)/n2

pa.total <- (sum(df.md1[which(df.md1$groups==”PIR”),"Probability_Model.1"]>0.5) + sum(df.md1[which(df.md1$groups==”AIR”),"Probability_Model.1]<0.5))/(n1+n2)

n1 # number of PIR in the data set of the model

n2 # number of AIR in the data set of the model

Table S1 | Measures computed in the first step of the clustering analysis.

| ***traj* package: Measures computed for of each trajectory** | |
| --- | --- |
| Measures | Formula |
| 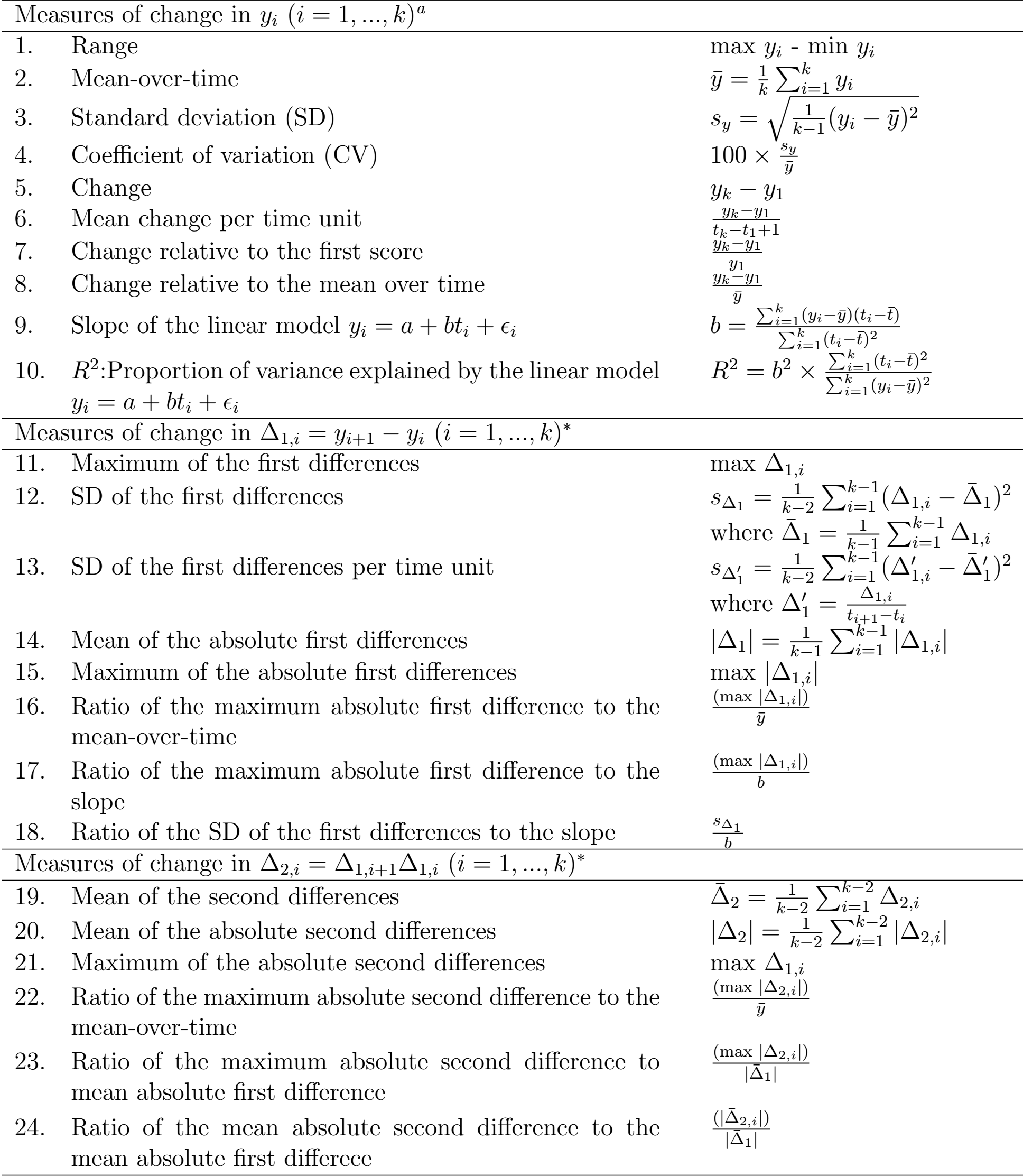 | |

Note: If a measure is equal to zero, it will be set to the smallest, non-zero value of the same measure across the sample during further calculations. If Y_1_, the first observation of the trajectory of an individual, is equal to zero, it will also be replaced.

Adapted from the *"User guides, package vignettes and other documentation"* section of R *traj package*.

Table S2 | R *traj* package clusters.

| **Cluster** | **Patient ID** | **CD4^+^ T cell count** (cells / µL)  at each time point (months on ART) | | | | | | | | | | | | | | |
| --- | --- | --- | --- | --- | --- | --- | --- | --- | --- | --- | --- | --- | --- | --- | --- | --- |
|  |  | **BL** | **2** | **6** | **9** | **12** | **16** | **20** | **24** | **28** | **32** | **36** | **42** | **48** | **54** | **60** |
| **Cluster 1**  **(*n* = 14)** | AH093 | 193 | 404 | 602 | 602 | 774 | 707 | 766 | 726 | 853 | 1044 | 896 | 986 | 902 | 1048 | 708 |
|  | AH083 | 181 | 325 | 353 | 362 | 508 | 467 | 768 | 589 | 503 | 612 | 551 | 562 | 624 | 613 | 619 |
|  | AH082 | 176 | 218 | 272 | 271 | 370 | 363 | 347 | 409 | 437 | 359 | 646 | NA | NA | NA | NA |
|  | AH092 | 164 | 461 | 310 | 429 | 628 | 401 | 514 | 428 | 456 | 378 | 424 | 519 | 586 | 589 | 599 |
|  | AH007 | 147 | 414 | 425 | NA | 534 | 819 | 629 | 679 | 732 | 694 | 681 | 846 | 621 | 828 | 727 |
|  | AH052 | 107 | 165 | 213 | 199 | 300 | 290 | 360 | 348 | 670 | 308 | 424 | 452 | 558 | 594 | 470 |
|  | AH095 | 94 | 308 | 222 | 358 | 253 | 266 | 319 | 341 | 397 | 582 | 397 | 488 | 323 | 512 | 505 |
|  | AH037 | 92 | 163 | 169 | 230 | 133 | 322 | 368 | 322 | 543 | 530 | NA | 534 | NA | NA | NA |
|  | AH033 | 68 | 270 | 282 | NA | 428 | 512 | 515 | 454 | 736 | 785 | 666 | NA | NA | NA | NA |
|  | AH044 | 56 | 489 | 463 | 621 | 537 | 505 | 434 | 439 | 488 | 560 | 660 | 599 | 442 | 518 | 736 |
|  | AH087 | 42 | 142 | 188 | 245 | 335 | 347 | 414 | 511 | 328 | 507 | 600 | NA | NA | NA | NA |
|  | AH053 | 32 | 244 | 256 | 111 | 518 | 475 | 526 | 337 | 482 | 520 | 501 | 609 | 692 | 796 | 825 |
|  | AH090 | 20 | 143 | 165 | 244 | 249 | 320 | 404 | 380 | 379 | 542 | 532 | 624 | 527 | 641 | 644 |
|  | AH066 | 12 | 112 | 155 | 186 | 209 | 274 | 475 | 375 | 405 | 537 | 436 | 565 | 473 | 595 | 526 |
| **Cluster 2**  **(*n* = 19)** | AH011 | 182 | 176 | 208 | NA | 198 | 220 | 301 | 304 | 287 | 260 | 309 | 319 | 275 | 255 | 261 |
|  | AH088 | 174 | 227 | 238 | 252 | 180 | 241 | 277 | 274 | 273 | 316 | 310 | 311 | 324 | 378 | 272 |
|  | AH099 | 161 | NA | 159 | 222 | 185 | 237 | 259 | 267 | 266 | 334 | 326 | 315 | 369 | 451 | NA |
|  | AH063 | 146 | 262 | 293 | 369 | 248 | 335 | 358 | 300 | 338 | 323 | 338 | NA | NA | NA | NA |
|  | AH089 | 143 | 396 | 254 | 256 | 328 | 240 | 304 | 263 | 290 | 261 | 325 | 343 | 338 | 223 | 347 |
|  | AH091 | 97 | 157 | 167 | 191 | 192 | 213 | 247 | 218 | 329 | 236 | 230 | 337 | 309 | 364 | NA |
|  | AH086 | 92 | 202 | 159 | 174 | 159 | 191 | 165 | 214 | 203 | 226 | 220 | 269 | 230 | 416 | 330 |
|  | AH100 | 67 | 102 | 109 | 173 | 172 | 170 | 348 | 287 | 458 | 325 | 378 | 392 | 494 | NA | NA |
|  | AH048 | 63 | 205 | 126 | 169 | 221 | 207 | 236 | 190 | 202 | 188 | 226 | 177 | 261 | 261 | 356 |
|  | AH074 | 61 | NA | 341 | 220 | 354 | 331 | 337 | 380 | 393 | 412 | 381 | NA | NA | NA | NA |
|  | AH079 | 47 | 142 | 166 | 145 | 253 | 176 | 226 | 307 | 341 | 344 | 290 | 351 | 326 | 296 | 393 |
|  | AH006 | 43 | 177 | 274 | NA | 252 | 304 | 367 | 363 | 403 | 328 | 355 | 247 | 299 | 230 | 310 |
|  | AH073 | 37 | 85 | 118 | 106 | 104 | 106 | 140 | 222 | 188 | 246 | 210 | 558 | 261 | 306 | 323 |
|  | AH013 | 30 | 60 | 190 | NA | 204 | 329 | 273 | 303 | 375 | 424 | 323 | NA | NA | NA | NA |
|  | AH035 | 25 | 229 | 178 | NA | 285 | 250 | 360 | 299 | 338 | 351 | 335 | 365 | 440 | NA | NA |
|  | AH003 | 22 | 135 | 128 | NA | 186 | 159 | 182 | 191 | 190 | 229 | 243 | 276 | 325 | 410 | 349 |
|  | AH080 | 14 | 167 | 197 | 152 | 197 | 263 | 327 | 302 | 327 | 320 | 392 | 381 | 395 | 447 | 548 |
|  | AH039 | 9 | 77 | 72 | 89 | 100 | 155 | 121 | 152 | 143 | 198 | 166 | 214 | 276 | 352 | NA |
|  | AH085 | 8 | 43 | 47 | 91 | 65 | 119 | 174 | 186 | 170 | 189 | 197 | 344 | 535 | 331 | 412 |

Note: Elements of each cluster are ordered by decreasing baseline CD4^+^ T cell count.

^a)^ Cells are shaded according to the CD4 count value: red, <200 CD4^+^ T cells/µL; orange, 200-350 CD4^+^ T cells/µL; yellow, 350-500 CD4^+^ T cells/µL; green, >500 CD4^+^ T cells/µL.

Abbreviations: BL, baseline (just before ART initiation); ID, identification code; NA, not available.

**Table S3 |** Signal joint TRECs and sj/β TREC ratio.

| **Cluster** | **Patient ID** | **sj-TREC** (number/10^5^ cells)  at each time point (months on ART) | | | | |  | **sj-TRECs** (number/mL whole blood)  at each time point (months on ART) | | | | |  | **sj/β TREC ratio** | | | | | |
| --- | --- | --- | --- | --- | --- | --- | --- | --- | --- | --- | --- | --- | --- | --- | --- | --- | --- | --- | --- |
|  |  | **BL** | **6** | **12** | **24** | **36** |  | **BL** | **6** | **12** | **24** | **36** |  | **BL** | **6** | **12** | **24** | **36** |  |
| **AIR**  **(*n* = 6)** | AH083 | 158,5 | 117,0 | 203,0 | 370,3 | 201,3 |  | 3186,1 | 3603,6 | 8098,6 | 10961,6 | 5837,9 |  | 15,9 | 23,7 | 13,9 | 42,8 | 39,6 |  |
|  | AH082 | 176,9 | 611,1 | 815,2 | 721,8 | 882,3 |  | 3556,5 | 13566,7 | 17772,4 | 15446,1 | 21440,9 |  | 30,1 | 42,2 | 57,9 | 114,4 | 67,3 |  |
|  | AH052 | 69,4 | 222,2 | 526,5 | 727,3 | 716,2 |  | 901,7 | 4222,0 | 9529,7 | 13455,3 | 11961,2 |  | 17,5 | 31,0 | 24,9 | 30,5 | 38,5 |  |
|  | AH033 | 57,3 | 628,6 | 1924,8 | 3084,0 | 3343,0 |  | 1214,2 | 9491,5 | 28872,7 | 47494,3 | 72209,3 |  | 1,9 | 31,5 | 131,5 | 37,4 | 35,8 |  |
|  | AH044 | 184,2 | 117,2 | 113,6 | 702,1 | 160,7 |  | 1657,2 | 5693,7 | 5999,9 | 17271,3 | 4900,7 |  | 54,1 | 23,1 | 17,2 | 103,9 | 8,6 |  |
|  | AH066 | 9,9 | 195,4 | 100,3 | 285,9 | 469,9 |  | 85,2 | 3634,0 | 1876,3 | 6518,1 | 10009,9 |  | 4,6 | 19,6 | 14,3 | 36,8 | 37,0 |  |
| **PIR**  **(*n* = 8)** | AH063 | 105,4 | 96,6 | 133,8 | 257,5 | 253,1 |  | 1739,3 | 1390,8 | 1498,2 | 3218,3 | 3214,9 |  | 21,6 | 19,0 | 30,9 | 17,3 | 18,3 |  |
|  | AH048 | 7,8 | 249,3 | 41,3 | 87,2 | 102,8 |  | 69,4 | 7554,6 | 1004,6 | 1787,1 | 1696,2 |  | 1,8 | 32,7 | 5,5 | 11,5 | 26,3 |  |
|  | AH074 | 32,1 | 110,6 | 171,2 | 258,4 | 227,6 |  | 568,3 | 3118,4 | 6180,8 | 7183,7 | 5463,1 |  | 14,7 | 30,0 | 44,3 | 25,5 | 24,2 |  |
|  | AH079 | 19,7 | 150,6 | 266,0 | 487,0 | 283,3 |  | 325,8 | 3313,2 | 6810,6 | 10325,1 | 5439,7 |  | 11,5 | 20,5 | 31,8 | 24,2 | 45,7 |  |
|  | AH073 | 85,7 | 306,8 | 191,1 | 232,1 | 204,4 |  | 1620,6 | 7487,0 | 2961,9 | 4851,0 | 3310,9 |  | 31,9 | 15,1 | 18,1 | 19,1 | 25,6 |  |
|  | AH035 | 190,1 | 354,1 | 197,3 | 512,4 | 641,2 |  | 2414,6 | 6514,9 | 4418,5 | 9479,4 | 13656,8 |  | 38,8 | 49,4 | 25,6 | 24,1 | 55,7 |  |
|  | AH080 | 2,3 | 28,2 | 51,4 | 135,4 | 132,4 |  | 17,5 | 423,7 | 745,1 | 2477,7 | 2622,0 |  | 0,8 | 3,0 | 15,9 | 13,8 | 17,9 |  |
|  | AH039 | 1,0 | 16,9 | 73,5 | 75,3 | 72,3 |  | 14,6 | 257,2 | 1175,4 | 1340,1 | 1562,6 |  | 0,3 | 4,2 | 15,7 | 4,7 | 20,7 |  |

Notes: AIR and PIR were separated based on clustering analysis of the longitudinal trajectories of CD4^+^ T cell counts, from baseline to 36 months of ART. Measurement of TRECs was only performed in a subgroup of patients. Elements of each group are ordered by decreasing baseline CD4^+^ T cell count.

Abbreviations: BL, baseline (just before ART initiation); ID, identification code; NA, not available; TRECs, T-cell receptor excision circles.

**Table S4 |** Absolute number and percentage of CD4^+^CD45RA^+^CD31^+^ T cells in peripheral blood.

| **Cluster** | **Patient ID** | **CD4 count** (cells / µL)  at baseline |  | **CD4^+^CD45RA^+^CD31^+^ T cells count** (cells / µL)  at each time point (months on ART) | | | | |  | **Percentage of CD31^+^CD45RA^+^ cells among CD4^+^ T cells** at each time point (months on ART) | | | | |
| --- | --- | --- | --- | --- | --- | --- | --- | --- | --- | --- | --- | --- | --- | --- |
|  |  |  |  | **BL** | **6** | **12** | **24** | **36** |  | **BL** | **6** | **12** | **24** | **36** |
| **Cluster 1**  **(*n* = 14)** | AH093 | 193,0 |  | 85,5 | 177,0 | 333,6 | 283,1 | 409,5 |  | 44,3 | 29,4 | 43,1 | 39,0 | 45,7 |
|  | AH083 | 181,0 |  | 34,4 | 79,4 | 119,9 | 123,1 | 120,7 |  | 19,0 | 22,5 | 23,6 | 20,9 | 21,9 |
|  | AH082 | 176,0 |  | 51,7 | 93,6 | 128,0 | 172,2 | 285,5 |  | 29,4 | 34,4 | 34,6 | 42,1 | 44,2 |
|  | AH092 | 164,0 |  | 6,3 | 32,6 | 80,4 | 51,8 | 39,1 |  | 3,8 | 10,5 | 12,8 | 12,1 | 9,2 |
|  | AH007 | 147,0 |  | 11,9 | 37,1 | NA | 134,4 | 178,4 |  | 8,1 | 8,7 | NA | 19,8 | 26,2 |
|  | AH052 | 107,0 |  | 8,2 | 36,4 | 91,5 | NA | 126,4 |  | 7,7 | 17,1 | 30,5 | NA | 29,8 |
|  | AH095 | 94,0 |  | 10,3 | 24,0 | NA | 74,0 | 101,2 |  | 11,0 | 10,8 | NA | 21,7 | 25,5 |
|  | AH037 | 92,0 |  | 9,9 | 26,0 | 21,8 | 135,2 | NA |  | 10,8 | 15,4 | 16,4 | 42,0 | NA |
|  | AH033 | 68,0 |  | 2,8 | 39,2 | 145,5 | 177,1 | 274,4 |  | 4,1 | 13,9 | 34,0 | 39,0 | 41,2 |
|  | AH044 | 56,0 |  | 13,4 | 75,0 | 66,6 | 77,7 | 123,4 |  | 24,0 | 16,2 | 12,4 | 17,7 | 18,7 |
|  | AH087 | 42,0 |  | 0,1 | 4,5 | 63,0 | 165,1 | 160,8 |  | 0,35 | 2,4 | 18,8 | 32,3 | 26,8 |
|  | AH053 | 32,0 |  | 10,6 | 43,0 | 166,8 | 115,9 | 167,8 |  | 33,0 | 16,8 | 32,2 | 34,4 | 33,5 |
|  | AH090 | 20,0 |  | 0,8 | 14,0 | NA | 171,0 | 212,3 |  | 4,2 | 8,5 | NA | 45,0 | 39,9 |
|  | AH066 | 12,0 |  | 0,2 | 25,9 | 39,3 | 103,9 | 103,3 |  | 1,6 | 16,7 | 18,8 | 27,7 | 23,7 |
| **Cluster 2**  **(*n* = 19)** | AH011 | 182 |  | 5,3 | 6,5 | 7,5 | 4,5 | NA |  | 2,9 | 3,1 | 3,8 | 1,5 | NA |
|  | AH088 | 174 |  | 3,6 | 1,9 | 6,8 | 12,4 | 11,7 |  | 2,1 | 0,8 | 3,8 | 4,5 | 3,8 |
|  | AH099 | 161 |  | 0,0 | 0,3 | 11,2 | 29,9 | 45,3 |  | 0,0 | 0,2 | 6,1 | 11,2 | 13,9 |
|  | AH063 | 146 |  | 19,9 | 84,7 | 45,4 | 46,8 | 61,9 |  | 13,6 | 28,9 | 18,3 | 15,6 | 18,3 |
|  | AH089 | 143 |  | 4,7 | 60,5 | 96,4 | 69,4 | 90,7 |  | 3,3 | 23,8 | 29,4 | 26,4 | 27,9 |
|  | AH091 | 97 |  | 4,9 | 24,7 | 51,5 | 73,5 | 71,5 |  | 5,1 | 14,8 | 26,8 | 33,7 | 31,1 |
|  | AH086 | 92 |  | 2,4 | 5,2 | 6,7 | 20,1 | 26,4 |  | 2,7 | 3,3 | 4,2 | 9,4 | 12,0 |
|  | AH100 | 67 |  | 2,7 | 17,0 | 41,3 | 82,1 | 96,4 |  | 4,1 | 15,6 | 24,0 | 28,6 | 25,5 |
|  | AH048 | 63 |  | 1,0 | 3,7 | 15,2 | 26,0 | 35,0 |  | 1,6 | 3,0 | 6,9 | 13,7 | 15,5 |
|  | AH074 | 61 |  | 17,7 | 64,4 | 92,7 | 105,6 | 81,9 |  | 29,0 | 18,9 | 26,2 | 27,8 | 21,5 |
|  | AH079 | 47 |  | 1,4 | 17,9 | 60,7 | 83,2 | 70,2 |  | 3,0 | 10,8 | 24,0 | 27,1 | 24,2 |
|  | AH006 | 43 |  | NA | NA | 1,5 | 4,8 | NA |  | NA | NA | 0,6 | 1,3 | NA |
|  | AH073 | 37 |  | 0,1 | 6,5 | 5,4 | 12,6 | 10,0 |  | 0,2 | 5,6 | 5,2 | 5,7 | 4,8 |
|  | AH013 | 30 |  | 6,4 | 11,9 | 24,1 | 76,1 | 73,6 |  | 21,3 | 6,3 | 11,8 | 25,1 | 22,8 |
|  | AH035 | 25 |  | 0,3 | 8,3 | 28,2 | 36,8 | 56,6 |  | 1,2 | 4,6 | 9,9 | 12,3 | 16,9 |
|  | AH003 | 22 |  | NA | NA | 1,3 | 3,3 | 6,2 |  | NA | NA | 0,7 | 1,7 | 2,6 |
|  | AH080 | 14 |  | 2,4 | 14,7 | 29,4 | 62,2 | 92,5 |  | 17,3 | 7,4 | 14,9 | 20,6 | 23,6 |
|  | AH039 | 9 |  | 0,3 | 2,7 | 6,5 | 10,1 | 24,4 |  | 3,3 | 3,8 | 6,5 | 6,7 | 14,7 |
|  | AH085 | 8 |  | NA | 1,0 | 1,2 | 17,0 | 47,3 |  | NA | 2,2 | 1,9 | 9,1 | 24,0 |

Notes: Clusters were formed based on the longitudinal trajectories of CD4^+^ T cell counts from baseline to 36 months of ART. Elements of each cluster are ordered by decreasing baseline CD4^+^ T cell count.

Abbreviations: BL, baseline (just before ART initiation); ID, identification code; NA, not available.

**Table S5** | Correlations between age and thymic function surrogates.

| **Correlation (Patient age, Parameter *X_i_*)** ^a)^ | | | | | | | |
| --- | --- | --- | --- | --- | --- | --- | --- |
| **Parameter *X_i_*** | **Time point** | **Total** | | **AIR** | | **PIR** | |
|  |  | Cor coeff ^b)^ | *p* | Cor coeff ^b)^ | *p* | Cor coeff ^b)^ | *p* |
| Thymic volume mean (cm^3^)  n = 16 (8 AIR, 8 PIR) | 0 M | *r* = 0.508 | **.045** | *r* = 0.690 | .058 | *r* = 0.235 | .575 |
|  | 12 M | *r* = 0.578 | **.019** | *r* = 0.698 | .054 | *r* = 0.534 | .172 |
| Thymic score (arbitrary units)  n = 16 (8 AIR, 8 PIR) | [0;12] M | *r_s_* = 0.334 | .206 | *r* = 0.489 | .219 | *r* = 0.314 | .449 |
| sj-TRECs (per 10^5^ cells)  n = 14 (6 AIR, 8 PIR) | 0 M | *r_s_* = - 0.186 | .525 | *r* = 0.085 | .873 | *r* = - 0.422 | .297 |
|  | 6 M | *r_s_* = 0.223 | .443 | *r_s_* = 0.232 | .658 | *r* = 0.130 | .758 |
|  | 12 M | *r_s_* = - 0.349 | .221 | *r* = - 0.153 | .772 | *r* = - 0.387 | .344 |
|  | 24 M | *r_s_* = - 0.144 | .624 | *r_s_* = - 0.145 | .784 | *r* = - 0.368 | .370 |
|  | 36 M | *r_s_* = - 0.175 | .551 | *r_s_* = 0.029 | .957 | *r_s_* = - 0.683 | .062 |
| sj/β TREC ratio  (arbitrary units)  n = 14 (6 AIR, 8 PIR) | 0 M | *r* = 0.110 | .709 | *r* = 0.433 | .391 | *r* = - 0.422 | .273 |
|  | 6 M | *r* = - 0.026 | .930 | *r* = 0.264 | .613 | *r* = - 0.239 | .569 |
|  | 12 M | *r_s_* = - 0.261 | .368 | *r_s_* = 0.232 | .658 | *r* = - 0.886 | **.003** |
|  | 24 M | *r_s_* = - 0.027 | .928 | *r_s_* = 0.406 | .425 | *r* = - 0.600 | .116 |
|  | 36 M | *r* = 0.087 | .768 | *r* = 0.128 | .809 | *r_s_* = 0.098 | .818 |
| Percentage of CD31^+^CD45RA^+^ cells among CD4^+^ T cells (%)  n = 33 (14 AIR, 19 PIR) | 0 M | *r_s_* = - 0.215 | .254 | *r_s_* = 0.121 | .680 | *r_s_* = - 0.271 | .309 |
|  | 6 M | *r_s_* = - 0.193 | .299 | *r* = 0.128 | .663 | *r_s_* = - 0.256 | .322 |
|  | 12 M | *r* = - 0.289 | .121 | *r* = - 0.025 | .941 | *r_s_* = - 0.148 | .545 |
|  | 24 M | *r* = - 0.280 | .121 | *r* = - 0.112 | .716 | *r* = - 0.116 | .635 |
|  | 36 M | *r* = - 0.285 | .126 | *r* = - 0.032 | .917 | *r* = - 0.252 | .329 |
| Absolute number of  CD31^+^CD45RA^+^ CD4^+^ T cells  (per / µL)  n = 33 (14 AIR, 19 PIR) | 0 M | *r_s_* = - 0.255 | .175 | *r_s_* = 0.053 | .858 | *r_s_* = - 0.198 | .463 |
|  | 6 M | *r_s_* = - 0.232 | .209 | *r_s_* = 0.095 | .747 | *r_s_* = - 0.273 | .290 |
|  | 12 M | *r_s_* = - 0.205 | .276 | *r_s_* = 0.128 | .708 | *r_s_* = - 0.104 | .672 |
|  | 24 M | *r_s_* = - 0.324 | .070 | *r* = - 0.313 | .298 | *r_s_* = - 0.120 | .623 |
|  | 36 M | *r_s_* = - 0.259 | .167 | *r* = - 0.119 | .699 | *r* = - 0.205 | .429 |

^a)^ Correlation between patient age at baseline (in years) and each of the parameters on the left column. ^b)^ Pearson or Spearman correlation coefficient (*r* or *r_s_*, respectively), according to Shapiro-Wilk test for normality assessment.

Abbreviations: Cor coeff, correlation coefficient; M, months on antiretroviral therapy; *p*, *p*-value.

**Table S6 |** Statistical comparison of multivariate logistic regression models.

|  | | **Logistic regression** | | | | | | | | |
| --- | --- | --- | --- | --- | --- | --- | --- | --- | --- | --- |
| **Models** | **Coefficients** | | **Predictor variables** | **LRT ^a)^** | **AIC ^b)^** | ***p-value* ^c)^** | **ROC analysis** | | |  |
|  |  |  |  |  |  |  | **AUC** | **ACC ^d)^** | **Sensitivity /**  **Specificity** |  |
| Model 1’  (*n*=28) | a = - 6.828  b = - 0.163  c = 2.117  d = 0.124 | | Age at baseline  Log HIV plasma VL at baseline  % RTE among CD4^+^ T cells at 6 mo | *p=*.193 | 31.946 | .047  .026  .060 | 0.878 | 78.6 % | 78.6 / 78.6% |  |
| Model 2’  (*n*=28) | a = -14.957  b = 2.391  c = 0.178  d = - 2.193 | | Log HIV plasma VL at baseline  CD4^+^ T cell count slope [0, 6] mo  % RTE among CD4^+^ T cells ratio _(0/6 mo)_ | *p=*.043^*^ | 30.279 | .081  .052  .044 | 0.872 | 78.6 % | 71.4 / 85.7% |  |
| Model 3’  (*n*=28) | a = -10.578  b = - 0.154  c = 2.211  d = 0.020 | | Age at baseline  Log HIV plasma VL at baseline  CD4^+^ T cell count at 6 mo | *p=*.654 | 27.857 | .046  .044  .047 | 0.918 | 82.1 % | 78.6 / 85.7% |  |
| Model 4’  (*n*=28) | a = - 9.720  b = - 0.160  c = 2.439  d = 0.011 | | Age at baseline  Log HIV plasma VL at baseline  CD4^+^ T cell count at 2 mo | *p=*.390 | 29.725 | .038  .018  .017 | 0.898 | 85.7 % | 78.6 / 92.9% |  |

Note: Models are ordered by increasing accuracy and specificity.

^a)^ Likelihood ratio test (LRT) was used to compare the goodness of fit of reduced model (3 final variables) *vs.* full model (initial variables); a non-significative *p*-value means that the reduced model is as good as the full model.

^b)^ AIC is an estimator of the relative quality of statistical models. Given two models for the same data set, the preferred model is the one with the minimum AIC value.

^c)^ *p*-value for each of the 3 predictors of the reduced model.

^d)^ Accuracy corresponds to the percentage of patients PIR or AIR that were respectively classified as “probably PIR” or “probably AIR” among all patients.

*) The full model has better goodness of fit than the reduced model considering α = .050.

Abbreviations: ACC, accuracy; AUC, area under the ROC curve; LRT, likelihood ratio test; mo, months; p, p-value; ROC, receiver operating characteristic curve; VL, viral load.

**Table S7** **|** “Probability of being PIR” among AIR and PIR, according to each multivariate logistic regression model.

| **Cluster** | **Patient ID** | **Age at BL** | **Log HIV plasma VL at BL** | **% RTE among CD4^+^ T cells at 6 mo** | **CD4^+^ T cell count slope [0, 6] mo** | **% RTE among CD4^+^ T cells ratio (0/6 mo)** | **CD4^+^ T cell count at 2 mo** | **CD4^+^ T cell count at 6 mo** | **“Probability of being PIR”** | | | |
| --- | --- | --- | --- | --- | --- | --- | --- | --- | --- | --- | --- | --- |
|  |  |  |  |  |  |  |  |  | **Model 1**  **(*n*=31)** | **Model 2**  **(*n*=30)** | **Model 3**  **(*n*=33)** | **Model 4**  **(*n*=31)** |
| **AIR**  **(*n* = 14)** | AH007 | 43 | 6,15 | 8,73 | 40,11 | 0,93 | 414,00 | 425,00 | 0,48 | 0,03 | 0,04 | 0,04 |
|  | AH033 | 33 | 6,52 | 13,90 | 31,00 | 0,29 | 270,00 | 282,00 | 0,09 | 0,01 | 0,05 | 0,02 |
|  | AH037 | 28 | 5,71 | 15,40 | 11,21 | 0,70 | 163,00 | 169,00 | 0,19 | 0,70 | 0,39 | 0,20 |
|  | AH044 | 46 | 5,25 | 16,20 | 57,21 | 1,48 | 489,00 | 463,00 | 0,78 | 0,08 | 0,18 | 0,22 |
|  | AH052 | 35 | 6,03 | 17,10 | 16,86 | 0,45 | 165,00 | 213,00 | 0,18 | 0,22 | 0,29 | 0,24 |
|  | AH053 | 51 | 6,98 | 16,80 | 32,43 | 1,96 | 244,00 | 256,00 | 0,15 | 0,08 | 0,17 | 0,12 |
|  | AH066 | 46 | 6,15 | 16,70 | 21,96 | 0,09 | 112,00 | 155,00 | 0,35 | 0,05 | 0,68 | 0,72 |
|  | AH082 | 51 | 5,34 | 34,40 | 16,82 | 0,85 | 218,00 | 272,00 | 0,42 | 0,80 | 0,78 | 0,93 |
|  | AH083 | 23 | 5,59 | 22,50 | 22,29 | 0,84 | 325,00 | 353,00 | 0,08 | 0,49 | 0,04 | 0,02 |
|  | AH087 | 32 | 6,42 | 2,38 | 17,63 | 0,15 | 142,00 | 188,00 | 0,26 | 0,05 | 0,16 | 0,09 |
|  | AH090 | 37 | 5,48 | 8,47 | 22,25 | 0,50 | 143,00 | 165,00 | 0,66 | 0,38 | 0,73 | 0,71 |
|  | AH092 | 42 | 5,33 | 10,50 | 10,88 | 0,36 | 461,00 | 310,00 | 0,78 | 0,75 | 0,47 | 0,15 |
|  | AH093 | 31 | 6,60 | 29,40 | 54,06 | 1,51 | 404,00 | 602,00 | 0,01 | 0,00 | 0,00 | 0,00 |
|  | AH095 | 36 | 5,90 | 10,80 | 14,25 | 1,02 | 308,00 | 222,00 | 0,38 | 0,66 | 0,34 | 0,09 |
| **PIR**  **(*n* = 19)** | AH003 | 44 | 5,41 | NA | 14,89 | NA | 135,00 | 128,00 | NA | NA | 0,91 | 0,91 |
|  | AH006 | 61 | 5,08 | NA | 37,30 | NA | 177,00 | 274,00 | NA | NA | 0,94 | 1,00 |
|  | AH011 | 36 | 4,90 | 3,11 | 4,86 | 0,93 | 176,00 | 208,00 | 0,91 | 0,99 | 0,80 | 0,86 |
|  | AH013 | 57 | 6,84 | 6,25 | 27,50 | 3,41 | 60,00 | 190,00 | 0,56 | 0,85 | 0,53 | 0,82 |
|  | AH035 | 37 | 5,29 | 4,64 | 20,04 | 0,26 | 229,00 | 178,00 | 0,81 | 0,44 | 0,76 | 0,58 |
|  | AH039 | 43 | 5,58 | 3,80 | 8,82 | 0,87 | 77,00 | 72,00 | 0,83 | 0,86 | 0,93 | 0,91 |
|  | AH048 | 47 | 5,56 | 2,97 | 6,18 | 0,55 | 205,00 | 126,00 | 0,89 | 0,82 | 0,91 | 0,82 |
|  | AH063 | 32 | 4,78 | 28,90 | 23,01 | 0,47 | 262,00 | 293,00 | 0,36 | 0,75 | 0,53 | 0,62 |
|  | AH073 | 41 | 4,95 | 5,55 | 11,68 | 0,03 | 85,00 | 118,00 | 0,92 | 0,77 | 0,95 | 0,97 |
|  | AH074 | 30 | 5,64 | 18,90 | 36,62 | 1,53 | NA | 341,00 | 0,19 | 0,37 | 0,09 | NA |
|  | AH079 | 41 | 5,03 | 10,80 | 17,44 | 0,28 | 142,00 | 166,00 | 0,85 | 0,69 | 0,90 | 0,94 |
|  | AH080 | 41 | 6,38 | 7,44 | 24,07 | 2,33 | 167,00 | 197,00 | 0,36 | 0,73 | 0,32 | 0,25 |
|  | AH085 | 28 | 4,94 | 2,17 | 5,28 | NA | 43,00 | 47,00 | 0,82 | NA | 0,93 | 0,89 |
|  | AH086 | 49 | 5,33 | 3,28 | 7,08 | 0,81 | 202,00 | 159,00 | 0,94 | 0,93 | 0,93 | 0,92 |
|  | AH088 | 67 | 5,60 | 0,80 | 4,80 | 2,60 | 227,00 | 238,00 | 0,99 | 1,00 | 0,95 | 0,99 |
|  | AH089 | 53 | 5,57 | 23,80 | 10,57 | 0,14 | 396,00 | 254,00 | 0,63 | 0,51 | 0,78 | 0,54 |
|  | AH091 | 48 | 6,33 | 14,80 | 5,36 | 0,34 | 157,00 | 167,00 | 0,37 | 0,33 | 0,61 | 0,56 |
|  | AH099 | 61 | 5,43 | 0,22 | 4,76 | 0,13 | NA | 159,00 | 0,98 | 0,76 | 0,97 | NA |
|  | AH100 | 44 | 4,87 | 15,60 | 5,46 | 0,26 | 102,00 | 109,00 | 0,86 | 0,94 | 0,97 | 0,98 |

Note: Cells are shaded in green if “Probability of being PIR” < 0.50 in AIR (true negatives) or ≥ 0.50 in PIR (true positives). Cells are shaded in red if “Probability of being PIR” ≥ 0.50 in AIR (false positives) or < 0.50 in PIR (false positives). Abbreviations: BL, baseline (just before ART initiation); ID, identification code; mo, months of ART; NA, not available.


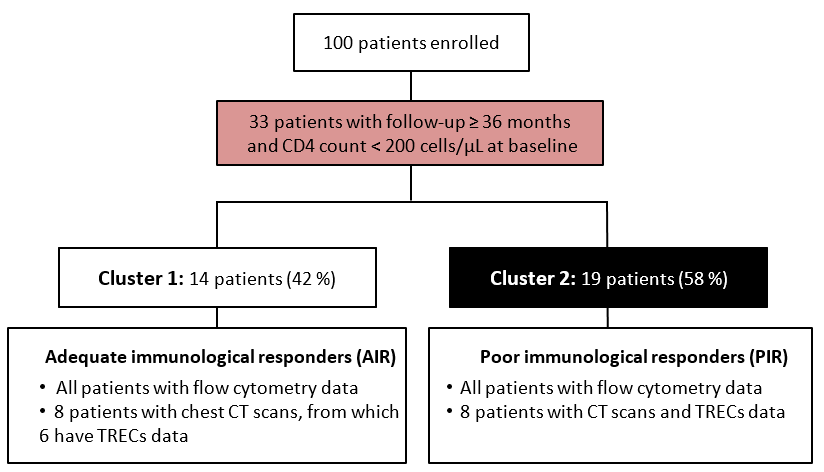


**Figure S1 | Patient selection and clustering process.** Thirty-three HIV-infected individuals, with CD4^+^ T cell counts over 200 cells/µL and with at least 36 months of follow-up since ART initiation, were selected from a prospective cohort of 100 individuals. Selected individuals were clustered according to their CD4^+^ count T cell trajectories during the 36 months of ART using *traj package* and classified as AIR (at least one CD4^+^ T cell count above 500 cells/µL during the first 36 months of ART) or PIR (all CD4^+^ T cell counts bellow 500 cells/µL).

**Figure S2 | Viral load levels throughout the follow-up period.** Each grey or blue line represents a single patient; blue lines stand for four patients who presented, each one, a virological blip, defined as single HIV RNA values between 50 and 400 copies/mL after 6 months of follow-up; the red line represents the median of all patients’ HIV plasma loads over time. The detection limit of the technique was 19 copies/mL of plasma [Log_10_(19)=1.28].


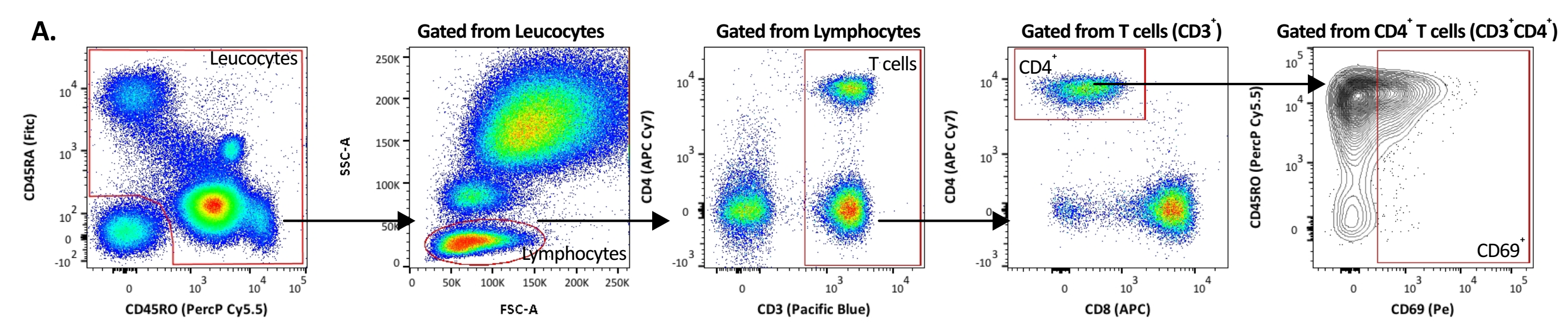

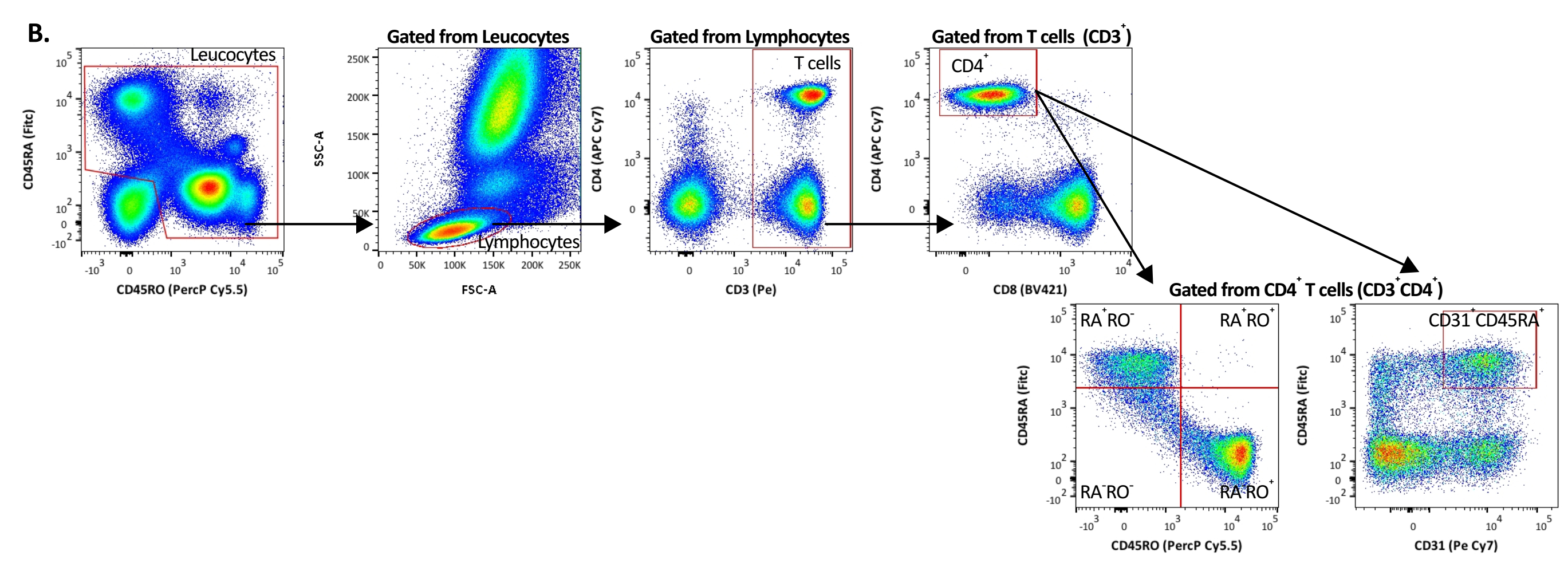


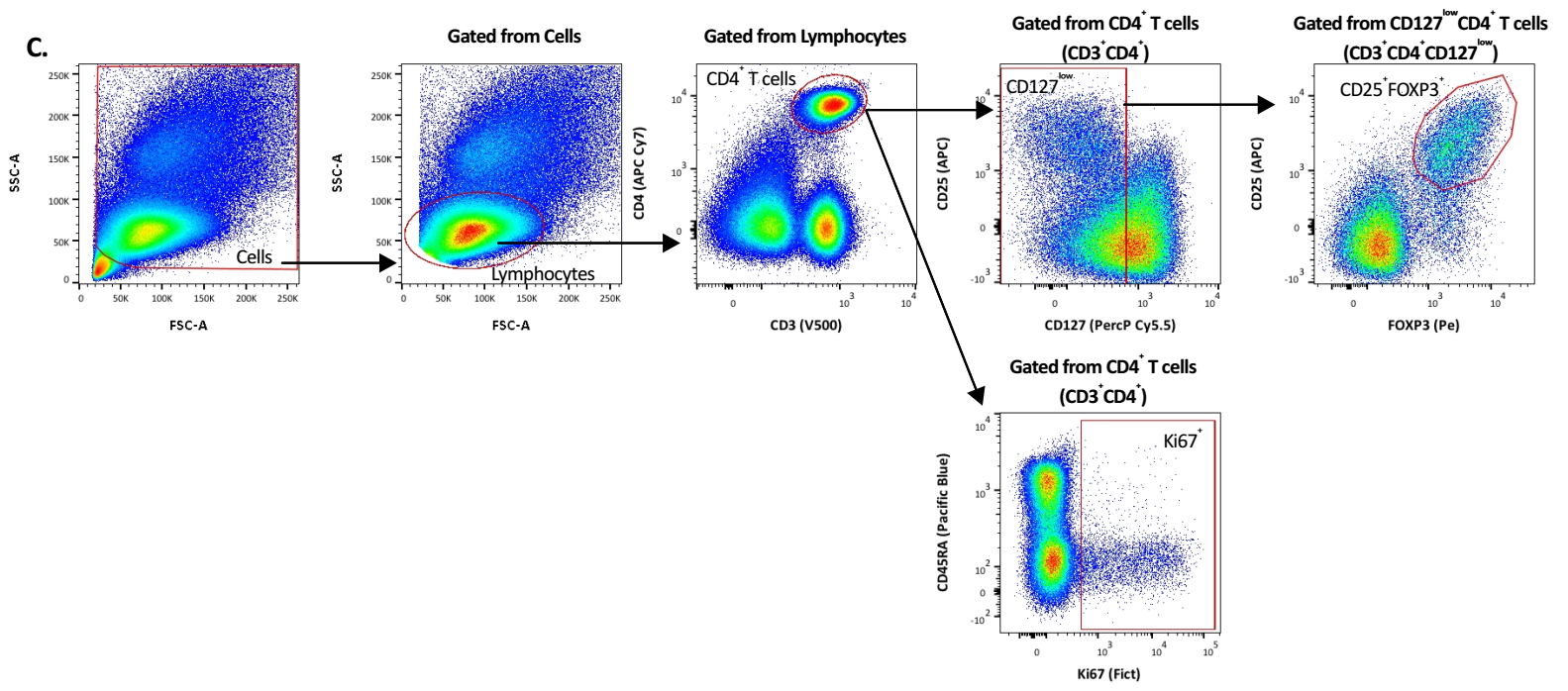


Figure S3 | Flow cytometry gating strategy. (A) Identification of CD69^+^ and HLA-DR^+^ (activation markers) cells among CD4^+^ T cells (panel 1); (B) Identification of CD45RA^-^CD45RO^+^ (memory), CD45RA^+^CD45RO^-^ (naïve) and CD45RA^+^CD31^+^ (recent thymic emigrants; RTE) cells among CD4^+^ T cells (panel 2). (C) Identification of CD3^+^CD4^+^CD127^low^CD25^high^FOXP3^+^ cells (regulatory T cells; Treg) and of Ki67^+^ (proliferation marker) cells among CD4^+^ T cells.

**
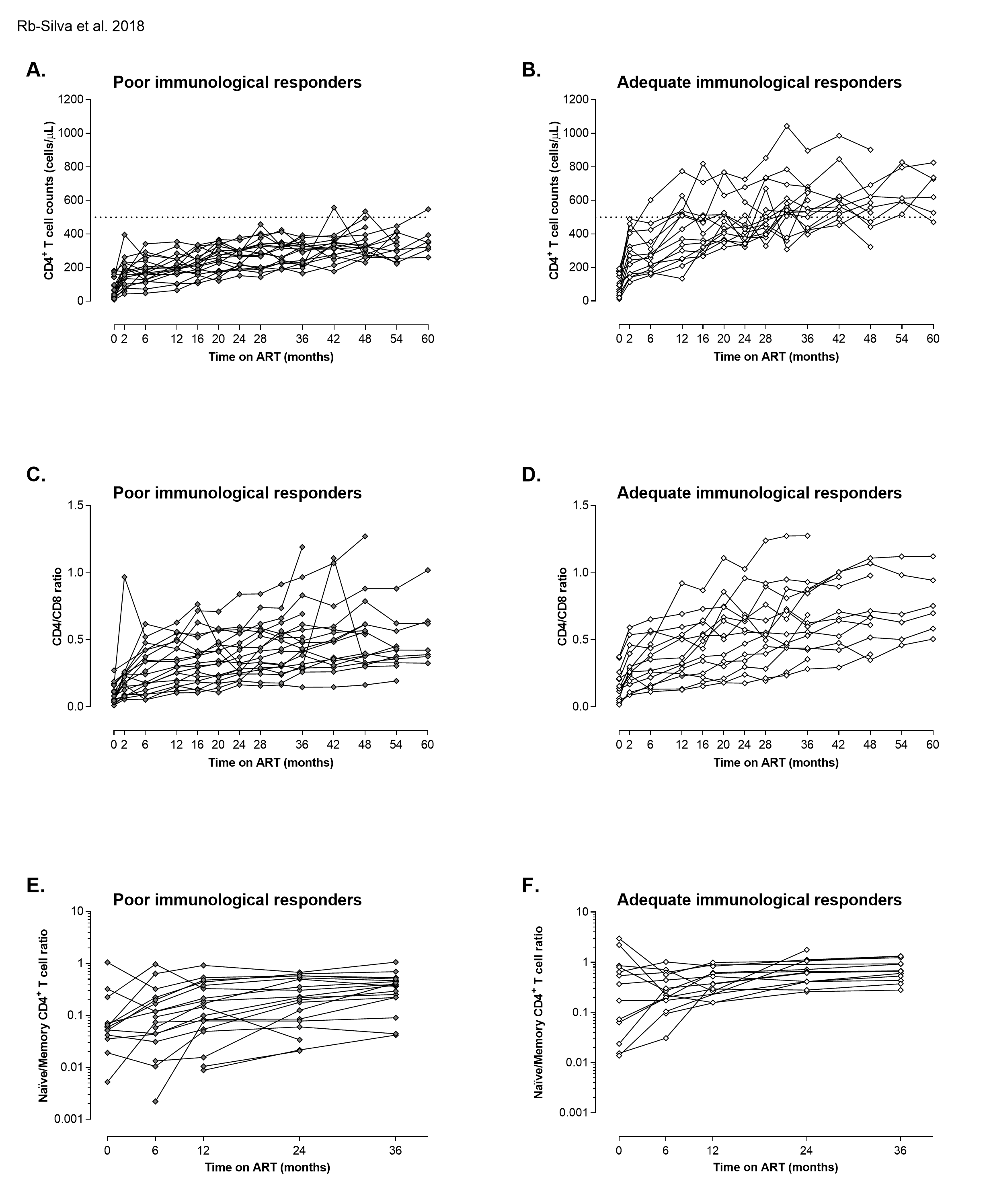
** **Figure S4 | Spaghetti plots. (A, B)** CD4^+^ T cell count evolution of each patient in PIR and AIR group, respectively. **(C, D)** CD4/CD8 ratio evolution of each patient in PIR and AIR group, respectively. **(E, F)** Naïve/Memory CD4^+^ T cell ratio evolution of each patient in PIR and AIR group, respectively.
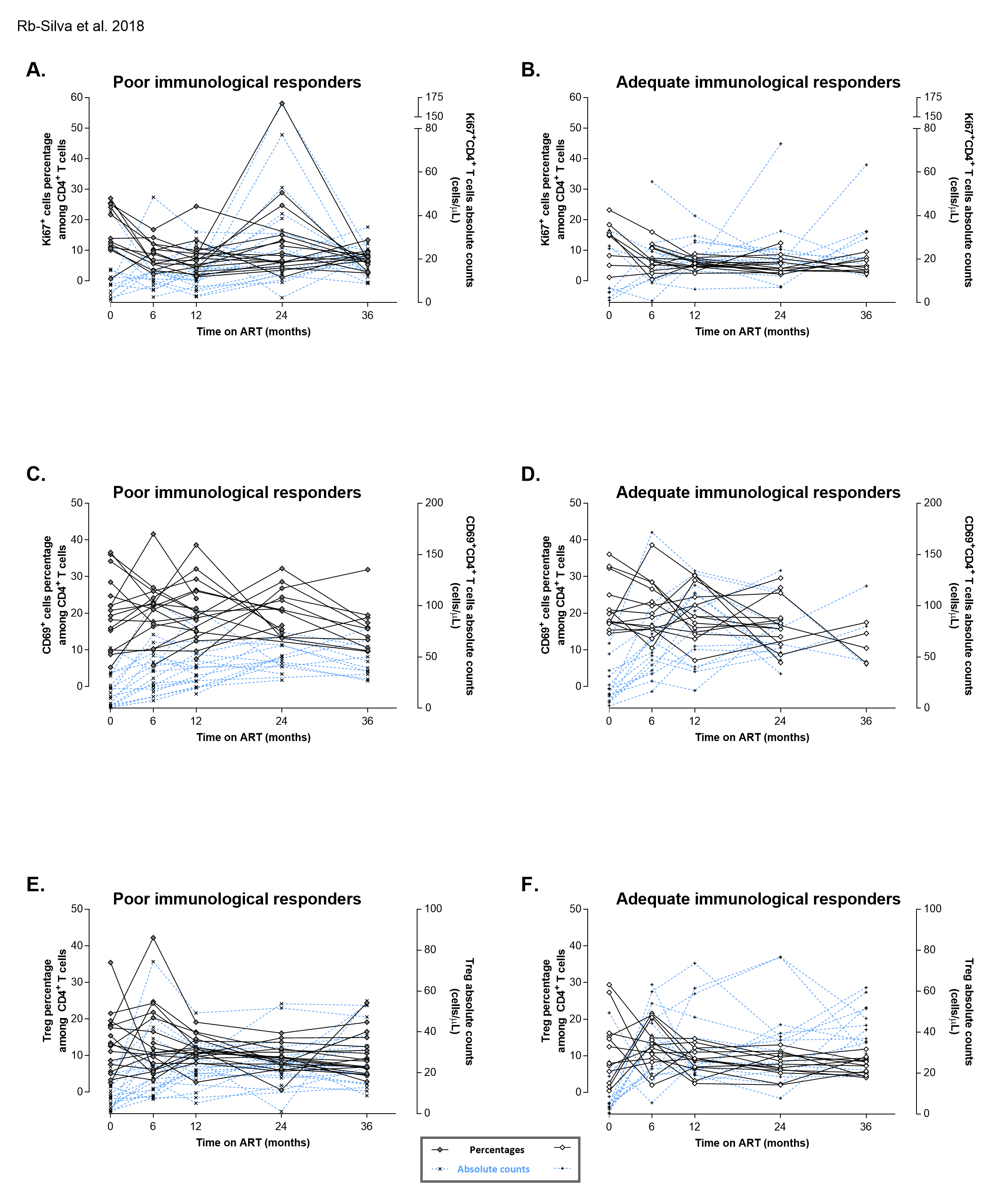
 **Figure S5 | Spaghetti plots. (A, B)** Percentage among CD4^+^ T cells and absolute number of Ki67^+^ cells in PIR and AIR group, respectively. **(C, D)** Percentage among CD4^+^ T cells and absolute number of CD69^+^ cells in PIR and AIR group, respectively. **(E, F)** Percentage among CD4^+^ T cells and absolute number of regulatory T cells (Treg) in PIR and AIR group, respectively. **
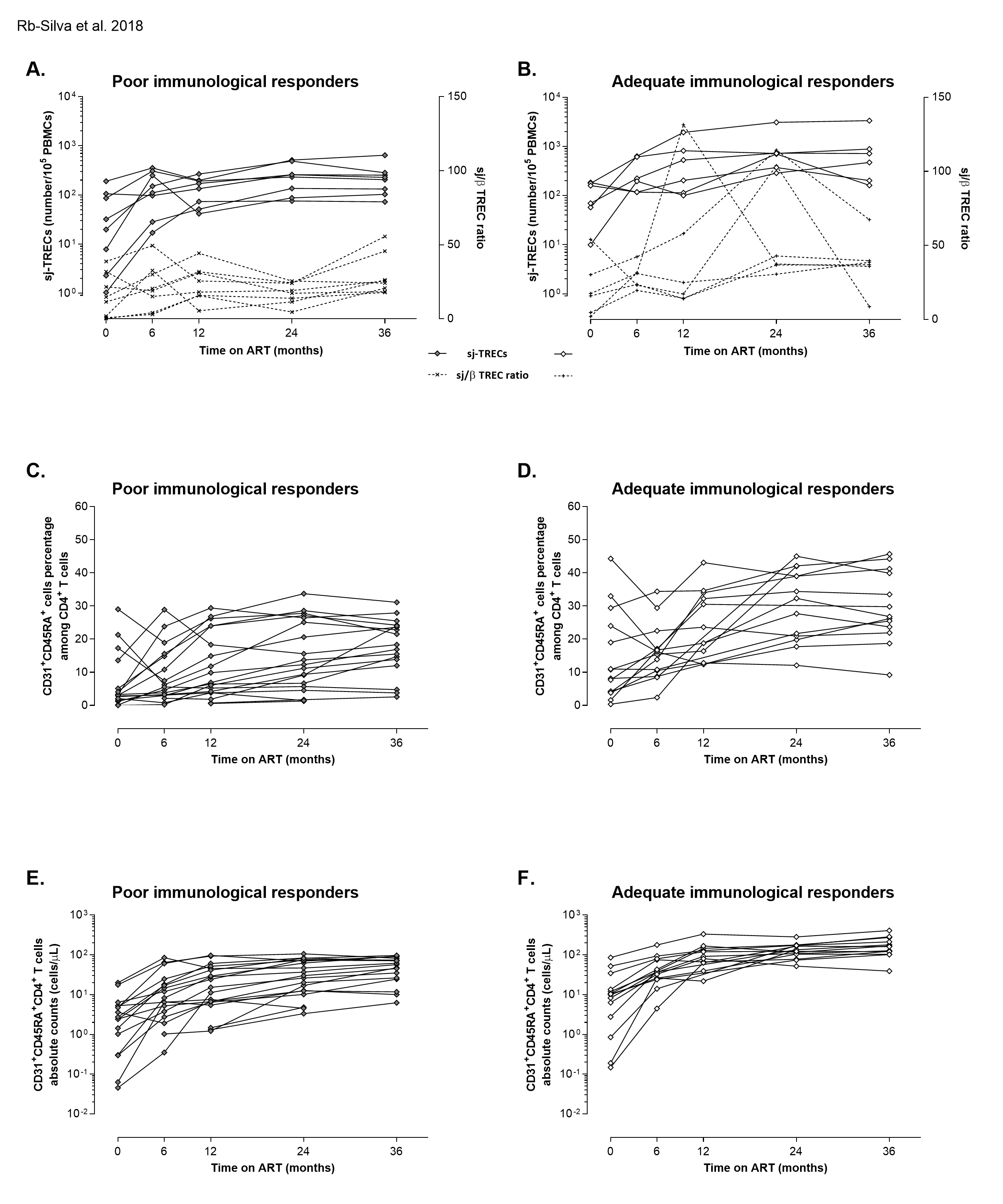
 Figure S6 | Spaghetti plots. (A, B)** Number of sj-TRECs in copies/10^5^ PBMCs and sj/βTREC ratio evolution for each patient in PIR and AIR group, respectively. **(C, D)** Percentage of CD31^+^CD45RA^+^ cells among CD4^+^ T cells evolution for each patient in PIR and AIR group, respectively. **(E, F)** Absolute number of CD31^+^CD45RA^+^CD4^+^ T cells evolution for each patient in PIR and AIR group, respectively.

**
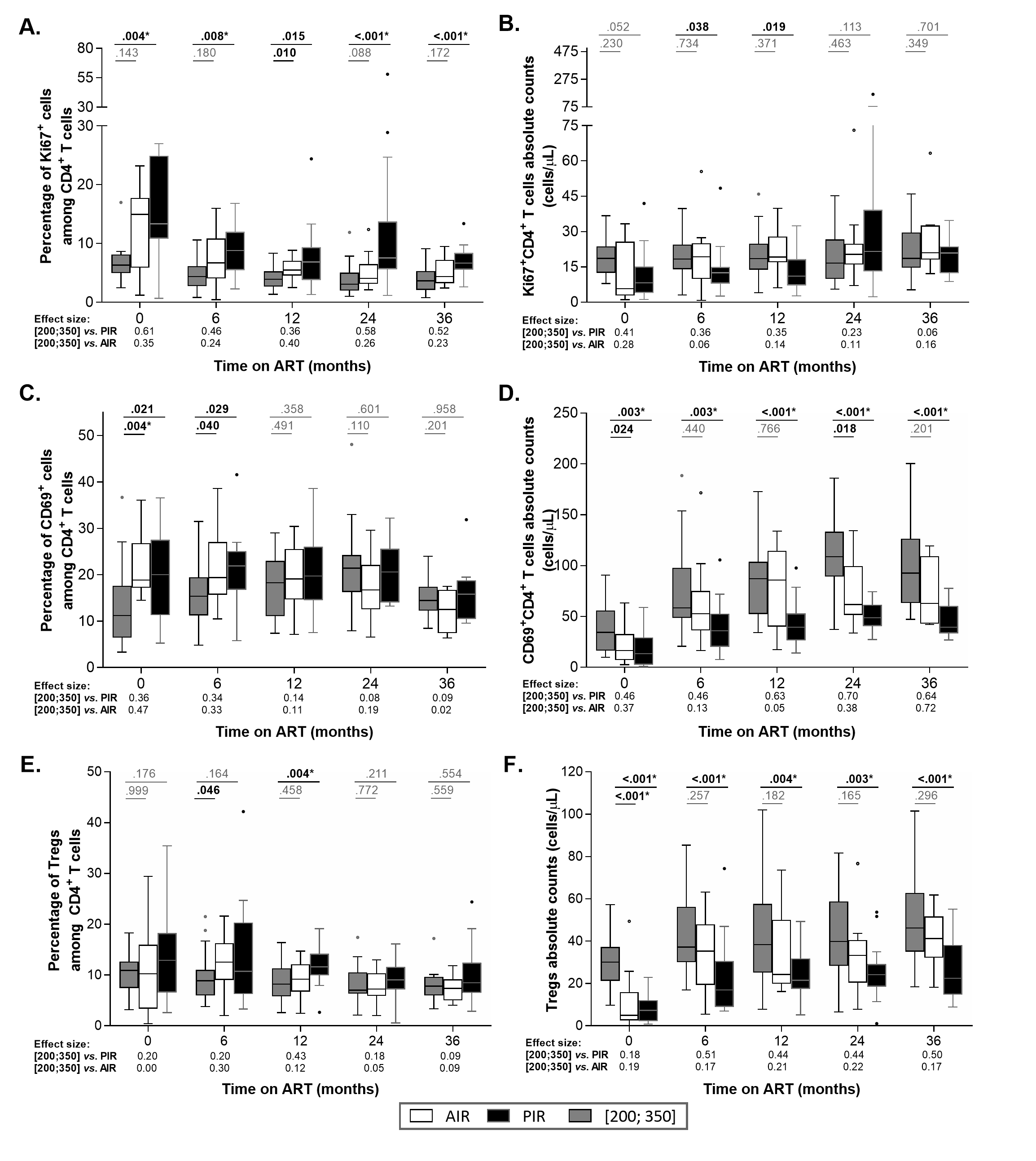
Figure S7 | Comparison of FACS data between patients with CD4^+^ T cell count of [200; 350] cells/µL and patients with CD4 count <200 cells/µL (AIR and PIR) at baseline, throughout antiretroviral therapy. (A)** Percentage of Ki67^+^ cells among CD4^+^ T cells; **(B)** Absolute numbers of Ki67^+^CD4^+^ T cells; **(C)** Percentage of CD69^+^ cells among CD4^+^ T cells; **(D)** Absolute numbers of CD69^+^CD4^+^ T cells; **(E)** Percentage of HLA-DR^+^ cells among CD4^+^ T cells; **(F)** Absolute numbers of HLA-DR^+^CD4^+^ T cells. Grey boxplots represent patients with [200; 350] cells/µL; white boxplots represent AIR; black boxplots represent PIR. Comparisons were performed using Wilcoxon/Mann-Whitney U-tests, and the correspondent r is presented as a measure of effect size. *, significant after Bonferroni correction (α / 5 = .010).

**
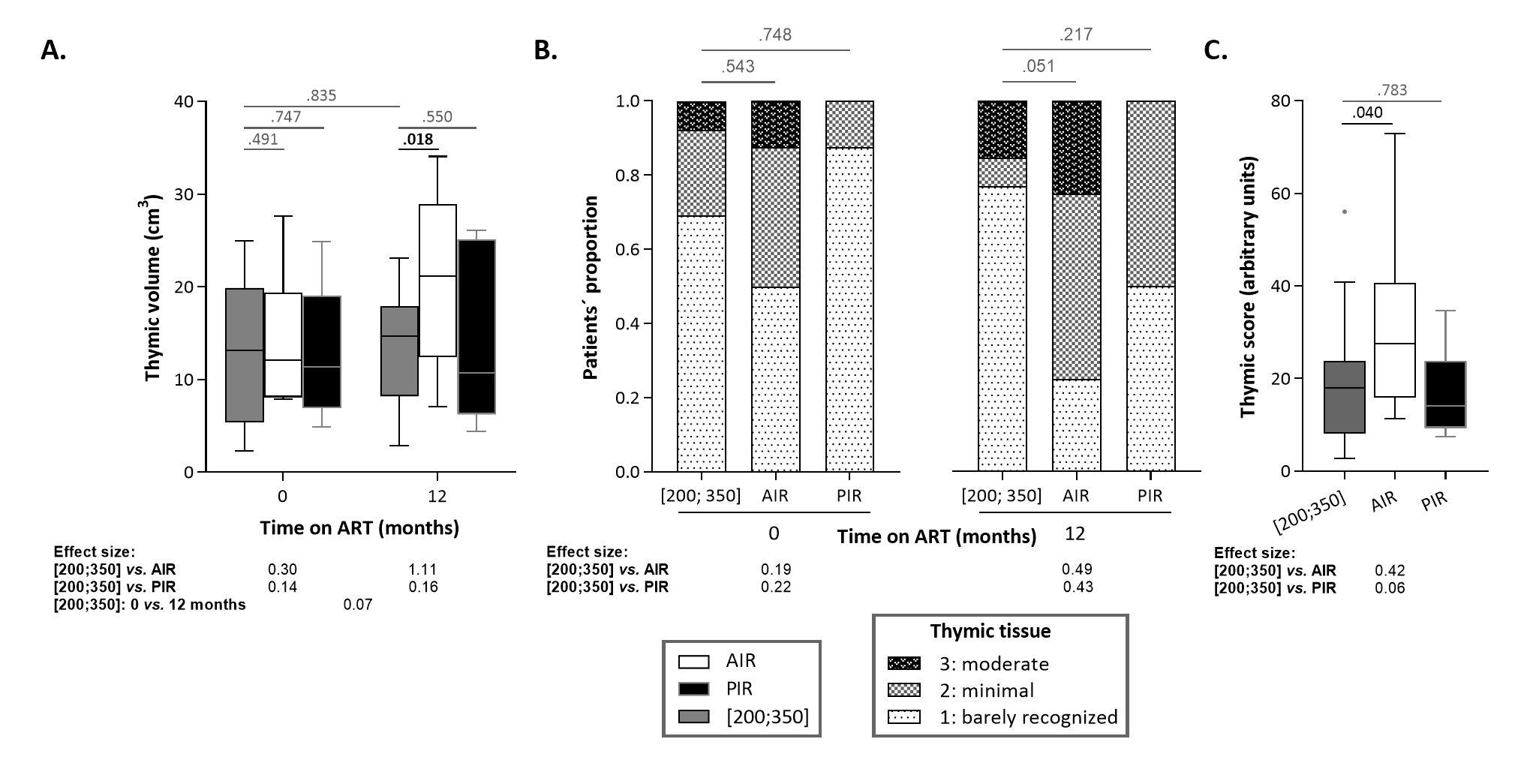
Figure S8 | Comparison of imaging data between patients with CD4^+^ T cell count of [200; 350] cells/µL and patients with <200 cells/µL (AIR and PIR) at baseline, throughout antiretroviral therapy. (A)** Thymic volume and **(B)** thymic index were compared between patients with CD4^+^ T count of [200; 350] cells/µL and AIR and PIR, respectively, at baseline and at 12 months of ART. **(C)** Thymic scores were calculated as the product of thymic volume and index means, over the first year of ART, and were also compared between those patients. Thymic volumes **(A)** were compared using independent t-tests; Cohen's d were calculated as a measure of effect size. Thymic indices **(B)** were compared using Fisher exact test and effect size estimates were calculated using Cramér’s V. Thymic scores **(C)** were compared using independent Wilcoxon-Mann-Whitney U-tests, and the correspondent r is presented as a measure of effect size.
